# Accelerated Pancreatic Volume Loss as a Potential Pre-Diagnostic Imaging Biomarker for Pancreatic Cancer Risk Enrichment: An AI-Assisted Volumetric Study

**DOI:** 10.3390/cancers18183062

**Published:** 2026-09-21

**Authors:** Jun Nakahodo, Wataru Ujita, Daichi Sadato, Yuki Fukumura, Yuji Aoki, Masataka Kikuyama, Shin-ichiro Horiguchi, Mizuka Suzuki, Kazuro Chiba, Hiroki Tabata, Kensuke Hoshi, Ryogo Minami, Toshiro Iizuka, Terumi Kamisawa, Masanao Kurata, Masakazu Toi

**Affiliations:** 1 Department of Gastroenterology, Tokyo Metropolitan Cancer and Infectious Diseases Center, Komagome Hospital, Bunkyo-ku, Tokyo 113-8677, Japan; wataru.ujita@med.toho-u.ac.jp (W.U.); kazurou_chiba@tmhp.jp (K.C.); hiroki.tabata.0108@gmail.com (H.T.); kensuke.hoshi@med.toho-u.ac.jp (K.H.); minami373323@gmail.com (R.M.); toshiro_iizuka@tmhp.jp (T.I.); terumi_kamisawa@tmhp.jp (T.K.); 2 Department of Human Pathology, Juntendo University, Bunkyo-ku, Tokyo 113-8431, Japan; yfuku@juntendo.ac.jp (Y.F.); yj-aoki@juntendo.ac.jp (Y.A.); 3 Clinical Research and Trials Center, Tokyo Metropolitan Cancer and Infectious Diseases Center, Komagome Hospital, Bunkyo-ku, Tokyo 113-8677, Japan; daichi_sadato@tmhp.jp; 4 Department of Gastroenterology, Tokyo Women’s Medical University Hospital, 8-1 Kawada-cho, Shinjuku-ku, Tokyo 162-8666, Japan; kikuyama110@yahoo.co.jp; 5 Department of Pathology, Tokyo Metropolitan Cancer and Infectious Diseases Center, Komagome Hospital, Bunkyo-ku, Tokyo 113-8677, Japan; horishin1@gmail.com; 6 Department of Radiology, Tokyo Metropolitan Cancer and Infectious Diseases Center, Komagome Hospital, Bunkyo-ku, Tokyo 113-8677, Japan; mizuka_suzuki@tmhp.jp; 7 Department of Hepato-Biliary-Pancreatic Surgery, Tokyo Metropolitan Cancer and Infectious Diseases Center, Komagome Hospital, Bunkyo-ku, Tokyo 113-8677, Japan; masanao_kurata@tmhp.jp; 8 Department of Breast Surgery, Tokyo Metropolitan Cancer and Infectious Diseases Center, Komagome Hospital, Bunkyo-ku, Tokyo 113-8677, Japan; masakazu_toi@tmhp.jp

**Keywords:** artificial intelligence, carcinogenesis, risk assessment, tumor microenvironment, linear mixed-effects model

## Abstract

**Background/Objectives:** Treatment for pancreatic ductal adenocarcinoma (PDAC) depends on early detection, yet most present at an inoperable stage. Focal pancreatic parenchymal atrophy (FPPA) is a prodromal imaging feature lacking objective quantification. We evaluated whether longitudinal, AI-measured pancreatic volume (PV) change is associated with subsequent PDAC. **Methods:** In this retrospective case–control study, we analyzed longitudinal CT from 40 PDAC patients and 61 controls, each with ≥5 years of pre-index imaging. PV was measured by AI segmentation and compared as a subject-specific rate of change, with robustness assessed via multivariable, mixed-effects, and sensitivity analyses. **Results:** PDAC patients showed more rapid PV decline than matched controls (5-year reduction 9.71 vs. 1.23 mL; *p* = 0.013), persisting after excluding diabetes and adjustment. A mixed-effects model confirmed faster decline, consistent across truncation windows but absent near diagnosis. Adding the marker to a basic covariate model raised the area under the curve (AUC) from 0.679 to 0.823. Exploratory transcriptomic profiling of microdissected FPPA identified 131 concordantly altered genes across PanIN- and invasive-PDAC-adjacent comparisons, with an immune-related signal present adjacent to non-invasive PanIN and larger adjacent to invasive PDAC. **Conclusions:** Patients who later developed PDAC showed greater long-term PV decline than controls. Exploratory tissue profiling raises the hypothesis that the same process underlying FPPA may also contribute to PV loss. Because the imaging and molecular analyses only partially overlapped and included no within-patient imaging–transcriptomic correlation, the proposed tissue-level correlate remains an unproven hypothesis requiring same-patient validation. This marker requires validation before use as a risk-enrichment signal, not a screening test.

## Simple Summary

Pancreatic cancer is usually found too late for cure because it causes no early symptoms. Using artificial intelligence, we automatically measured pancreas size on CT scans taken years before diagnosis in 40 patients who later developed pancreatic cancer and 61 who did not develop pancreatic cancer. Those who later developed cancer showed faster shrinkage of the pancreas, detectable more than a year before diagnosis. The difference persisted across several complementary analyses, including a model accounting for each patient’s individual scanning schedule, and did not depend on diabetes status or other measurable clinical factors. In tissue from a partly overlapping group of patients, immune- and remodeling-related changes were observed adjacent to high-grade PanIN and invasive PDAC. Because no patient-level link between imaging and tissue was examined, these findings provide biological plausibility but do not establish the mechanism of CT-detected volume loss. This marker cannot replace a cancer diagnosis and requires validation in larger studies, but automated tracking of pancreas volume on scans performed for other reasons could help flag individuals who warrant closer monitoring for pancreatic cancer.

## 1. Introduction

Pancreatic ductal adenocarcinoma (PDAC) is associated with a high mortality rate, with 70–80% of cases diagnosed at inoperable stages. Although surgical resection offers the only chance for cure, the 5-year relative survival rate remains low at 13.7%, varying significantly by stage: 3.4% for distant metastases, 17.0% for regional disease, and 43.6% for localized tumors [1]. Early detection is therefore the most effective strategy to improve treatment outcomes. While the median survival for high-risk individuals detected via surveillance is 9.8 years compared to 1.5 years for symptomatic patients [2], the changing epidemiology of PDAC and recent advances in risk stratification, imaging, and biomarker-based detection have reshaped strategies for identifying patients most likely to benefit from early intervention [3,4,5]. Nonetheless, broad population-based screening is not currently recommended because the low incidence of PDAC requires extremely high specificity to avoid numerous false positives [6,7]. Accordingly, biomarkers in this clinical setting are more realistically conceived not as stand-alone diagnostic or screening tests but as tools for prognostic risk enrichment—identifying a subset of individuals at meaningfully higher risk who warrant closer surveillance—a distinction conceptually analogous to the ‘prognostic enrichment’ strategies formalized in the clinical-trial-design literature for selecting higher-risk populations [8,9], although the present application is to real-world surveillance triage rather than trial enrollment.

Focal pancreatic parenchymal atrophy (FPPA) has emerged as a potential prodromal feature that precedes PDAC diagnosis by 1–5 years [10,11,12,13]. Histologically, FPPA may be associated with high-grade pancreatic intraepithelial neoplasia (PanIN); progressive FPPA without accompanying main pancreatic duct change has been reported as the sole imaging clue to an underlying carcinoma in situ [14], and FPPA has been linked to high-grade PanIN across several dedicated series [15,16,17], while diffuse pancreatic atrophy is also linked to an elevated risk of PDAC [18]. Although FPPA denotes localized parenchymal loss, cumulative focal atrophy contributes to a measurable reduction in whole-gland volume; total pancreatic volume, which can be quantified automatically and reproducibly from routine CT, therefore provides a practical whole-organ surrogate for the aggregate atrophic process, albeit without spatial localization of the affected region. These atrophic patterns have been further stratified into focal, upstream duct-obstruction-related, and diffuse subtypes with distinct etiologies, imaging appearances, and surgical implications [19]. Distinguishing such pre-invasive, atrophy-associated lesions from small invasive PDAC remains clinically important, as the two entities differ substantially in prognosis. The early microenvironment of PanIN lesions exhibits epithelial plasticity and stromal cell recruitment, promoting disease progression [20]. However, the clinical utility of FPPA is currently limited by substantial heterogeneity in reported detection rates. Studies specifically designed to characterize atrophy in cohorts with confirmed early-stage or small PDAC, carcinoma in situ, or high-grade PanIN have reported FPPA frequencies of 40.6–83.3% (mean approximately 62%) [15,16,21,22,23,24,25,26], whereas studies that retrospectively reviewed imaging obtained before a subsequent clinical diagnosis of PDAC report substantially lower frequencies of 26.6–37.9% (mean approximately 32%) [10,11,12,13]. This roughly two-fold discrepancy likely reflects at least three factors: the early-stage cohorts were, by design, already selected for resection or biopsy on the basis of indirect imaging or cytological findings, which enriches for radiologically overt atrophy in a way that cannot be extrapolated to an unselected pre-diagnostic population; no internationally unified definition or measurement protocol for FPPA yet exists, so studies differ in what is counted as a positive finding; and single-time-point visual assessment is inherently constrained by substantial inter-individual variation in normal pancreatic morphology, such that formal inter-observer agreement for FPPA has not been established. This heterogeneity underscores the need for objective, quantifiable, longitudinal tools, rather than a single subjective visual read, to stratify patients for early intervention.

AI-based analysis of medical imaging, electronic health records, and liquid-biopsy biomarkers is increasingly used to surface weak, distributed pre-diagnostic signals that precede overt radiologic findings in PDAC [27]. Unlike conventional single-time-point visual assessments of focal pancreatic atrophy, this study introduces an AI-assisted volumetric framework that quantifies long-term, within-person pancreatic volume trajectories to objectively assess prodromal atrophy before PDAC diagnosis. Furthermore, as a pilot investigation of the biological background of these radiological changes, we performed exploratory molecular analyses of laser-microdissected atrophic acinar regions adjacent to high-grade PanIN or small invasive PDAC, together with a separate analysis of neoplastic epithelium across PanIN and invasive carcinoma. This design allowed us to examine whether FPPA represents only passive acinar loss or, instead, a biologically active parenchymal state in which acinar remodeling and immune activation proceed in parallel with neoplastic epithelial progression—a two-compartment view of early pancreatic carcinogenesis in which the peri-lesional acinar parenchyma, and not only the neoplastic epithelium, evolves during tumor development [28,29,30]. By integrating AI-based imaging with these exploratory tissue-level observations, and by subjecting the primary finding to multiple independent robustness checks, we aim to provide a rationale for future large-scale prospective studies validating rapid PV loss as a marker to inform pancreatic surveillance intensity. Because the marker relies on serial imaging accumulated over years, its realistic near-term application is opportunistic—computed automatically from CT already obtained for unrelated indications and stored in the picture archiving and communication system (PACS)—rather than as a dedicated screening test; this positions it as a risk-enrichment step feeding existing surveillance pathways.

## 2. Materials and Methods

### 2.1. Study Design and Ethics

This retrospective observational study was conducted in accordance with the principles of the Declaration of Helsinki and was approved by the Institutional Review Board (IRB) of Komagome Hospital (IRB number: 2721; approved 28 May 2021). The requirement for written informed consent was waived due to the opt-out nature of the study design. Information regarding the study was publicly disclosed on the hospital website, providing patients with the opportunity to decline participation.

### 2.2. Patient Selection and Matching

We reviewed 1916 pancreatic pathology specimens (492 surgical and 1424 endoscopic ultrasound-guided fine-needle aspiration samples), obtained during the study period (2015–2023), representing 1129 unique cases with a pancreatic malignancy. Forty patients with PDAC who had available prediagnostic imaging were identified. Exclusion criteria were: (1) intraductal papillary mucinous neoplasm (IPMN)-derived PDAC, (2) prior chemotherapy, (3) fewer than three CT scans available, and (4) a retrospective observation period of less than 5 years. No formal a priori sample-size or power calculation was performed; all patients with PDAC meeting the eligibility criteria during the study period were included, and the final sample size was therefore determined by the number of eligible cases available for retrospective analysis rather than by a pre-specified target.

**Figure 1:**
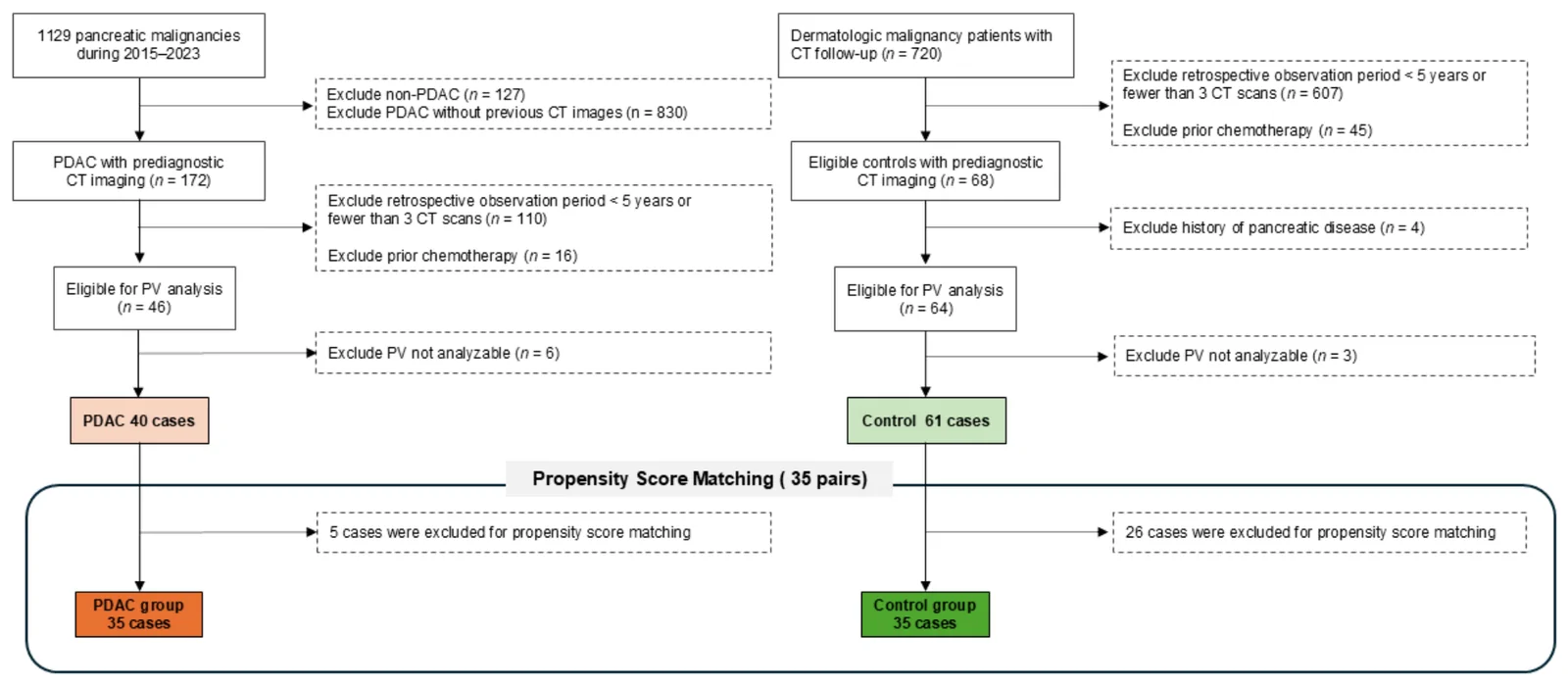
Flow diagram of case selection and propensity score matching.

Control patients were selected because ongoing oncologic CT surveillance provided serial pre-diagnostic imaging suitable for within-person longitudinal volumetry, which is not available for healthy volunteers. The controls included 61 patients with dermatologic malignancies (malignant melanoma, *n* = 23; Paget disease, *n* = 14; squamous cell carcinoma, *n* = 10; sweat gland carcinoma, *n* = 6; dermatofibrosarcoma, *n* = 3; Merkel cell carcinoma, *n* = 2; basal cell carcinoma, *n* = 2; sebaceous gland carcinoma, *n* = 1) who had no history of pancreatic disease, chemotherapy, or abdominal surgery, and who underwent at least three CT scans over a period of ≥5 years. Controls were not healthy individuals or a PDAC-enriched high-risk group, and differences in systemic inflammatory burden, metabolic status, body composition, and healthcare utilization relative to the PDAC group cannot be excluded and are considered in Section 4. Following propensity score matching for age and sex, 35 pairs were assigned to the PDAC and control groups (Figure 1). Covariate balance before and after matching was assessed using standardized mean differences (SMDs), with |SMD| < 0.10 regarded as adequate balance (Supplementary Table S7); matching balanced age and sex (|SMD| ≈ 0), whereas the number of CT scans (SMD −0.99) and several comorbidities showed residual imbalance and were addressed analytically by multivariable adjustment and by the linear mixed-effects model.

### 2.3. CT Volumetry and Image Analysis

CT images were obtained as part of routine clinical care using heterogeneous real-world imaging protocols, including both contrast-enhanced and non-contrast examinations. PV was assessed under this variability to reflect clinical practice, with image data standardized to 5 mm slices for analysis. PV was automatically calculated using the AI-assisted 3D analysis software, SYNAPSE VINSENT cloud. (Fujifilm, Japan) (Supplementary Figure S1).

All automated segmentations were visually inspected on axial, coronal, and sagittal reconstructions; scans on which the pancreas could not be reliably delineated were excluded (Figure 1). In total, 61 examinations were excluded at this quality-control step: in the PDAC group, 19 scans (7 unenhanced and 12 contrast-enhanced CT) on which the pancreas could not be reliably measured (e.g., owing to cystic change or pancreatitis); and in the control group, 42 scans (21 unenhanced and 21 contrast-enhanced CT) on which the automated segmentation extended beyond the pancreatic margin or underestimated the gland. The pancreatic-segmentation performance of this deep-learning engine has been independently validated against expert manual reference, with a reported pancreatic-parenchyma Dice similarity coefficient of 0.84 (0.83 in patients with pancreatic cancer and 0.86 in non-cancer controls) [31]. In contrast to classical texture- and entropy-based image-segmentation methods, in which anatomical boundaries are delineated from hand-crafted statistical texture descriptors [32], this deep-learning approach learns organ appearance directly from annotated images and requires only operator verification, reducing operator dependence in delineating the pancreas. Segmentation itself was fully automated by the AI software(SYNAPSE VINSENT cloud) and was not manually corrected; two observers divided the case list between them and each performed visual plausibility screening and recorded the resulting volumes for their assigned cases, without both observers independently reviewing the same cases. To validate measurement consistency, Pearson correlation and Bland–Altman analyses were performed on 134 paired contrast-enhanced and unenhanced CT images. The PDAC diagnosis date was defined as the date of the diagnostic CT scan confirming PDAC. PV ratios were calculated relative to the “Initial PV,” defined as the earliest PV measured >5 years before diagnosis.

### 2.4. Histopathological and Molecular Analyses

Histopathological assessment (PanIN prevalence) was restricted to the nine patients who underwent surgical resection (Supplementary Table S1). PanIN prevalence was calculated for each case as the proportion of PanIN-positive slides relative to the total parenchymal tissue slides, excluding invasive PDAC areas; group-level values are reported as the mean of these per-case prevalences. For exploratory molecular analysis, we selected six patients who had not undergone preoperative chemotherapy: three patients diagnosed after 2020 with a small invasive PDAC (Cases 7–9 in Supplementary Table S1), and three additional patients with high-grade PanIN but no invasive carcinoma, who were not among the nine patients in Supplementary Table S1. RNA was extracted from formalin-fixed paraffin-embedded (FFPE) tissues. Using laser capture microdissection, 12 regions were isolated in total: from each of the three invasive-PDAC patients (Cases 7–9), one atrophic acinar area near the small invasive PDAC (FPPA-invasive) and one adjacent normal-appearing acinar area (normal-invasive) were isolated; from each of the three high-grade-PanIN patients, one atrophic acinar area near the high-grade PanIN (FPPA-high) and one adjacent normal-appearing acinar area (normal-high) were isolated—yielding three FPPA-high, three FPPA-invasive, three normal-high, and three normal-invasive regions in total, two regions from each of the six patients. Because each patient contributed only a single region per lesion category, no individual was counted more than once within any given region-level comparison, avoiding pseudo-replication in the differential-expression screen. This sampling strategy was designed to capture microenvironment-dependent phenotypic differences, given evidence that invading tumor cells preferentially colonize inflamed, injured pancreatic lobules marked by activated, damage-responsive fibroblasts and distinct immune infiltrates [30]. RNA expression was analyzed using the nCounter Tumor Signaling 360 panel (NanoString Technologies, Seattle, WA, USA; 780 target genes plus internal reference genes) and processed with nSolver 2.6 software. Counts were normalized in three sequential steps: positive-control normalization using the geometric mean of the spike-in positive-control probes; background correction using the mean plus two standard deviations (SD) of the negative-control probes; and reference-gene (housekeeping) normalization using the geometric mean of stably expressed housekeeping genes. The housekeeping genes were selected from the panel’s built-in reference candidates by ranking their expression stability with the geNorm algorithm; the 17 most stable candidates were retained for normalization (TMUB2, ERCC3, NRDE2, PUM1, SF3A1, TLK2, ABCF1, SDHA, TBP, MRPL19, GUSB, TBC1D10B, STK11IP, POLR2A, G6PD, PSMC4, and DNAJC14), whereas three unstable candidates (UBB, TFRC, and OAZ1; SD after normalization ≥ 0.4) were discarded (Supplementary Table S6). After background thresholding, 669 transcripts were retained for differential expression analysis. All fold changes, confidence intervals, and adjusted *p* values reported in this manuscript, including those underlying the volcano plots and supplementary gene tables, derive from a single differential expression output generated by this pipeline. Gene-level biological annotations and functional themes used for interpretation were those provided with the panel. Differential expression was assessed for two lesion-versus-normal comparisons: FPPA-high versus normal-high, and FPPA-invasive versus normal-invasive, with the Benjamini–Yekutieli (BY) procedure applied to control the false discovery rate, chosen because it remains valid under arbitrary dependency among tests, as is expected for co-regulated transcripts [33]; BY-adjusted *p* < 0.05 was considered significant. To assess whether findings reflected a consistent biological signal rather than small-sample noise, we additionally examined the overlap and directional concordance of significantly differentially expressed genes across these two comparisons. Each comparison used three lesion regions and three normal regions; because the FPPA-high/normal-high comparison and the FPPA-invasive/normal-invasive comparison were performed on tissue from two non-overlapping sets of three patients, the two comparisons are independent of one another, although each remains limited by its very small per-comparison sample size, as discussed further in the Limitations.

In addition to the FPPA and adjacent normal-appearing acinar regions described above, laser capture microdissection also isolated the corresponding epithelial lesions from the same tissue blocks: low-grade PanIN epithelium from all six patients, high-grade PanIN epithelium from the three high-grade-PanIN patients, and invasive PDAC epithelium from the three small-invasive-PDAC patients, yielding 12 additional regions (24 regions in total across the six patients). These regions were processed and analyzed with the same nCounter panel and background-thresholding pipeline described above. The epithelial-lesion regions were then used to test whether molecular differences associated with a precursor-to-invasive transition could be detected within the microdissected epithelial-lesion compartment, using an additional, histologically intermediate-to-advanced comparison. Low-grade and high-grade PanIN epithelium were pooled into a single PanIN-epithelium group (nine regions from all six patients) and compared against the invasive PDAC epithelium group (three regions, from the three small-invasive-PDAC patients). Because the three high-grade-PanIN patients each contributed both a low-grade and a high-grade PanIN-epithelium region, and are therefore represented twice within the pooled PanIN group, this comparison was analyzed using a linear mixed-effects model with a patient-level random intercept to account for within-patient correlation among all repeated epithelial regions contributed by the same patient, rather than treating individual regions as statistically independent observations; fixed-effect *p* values for the group contrast were adjusted across the 669 tested transcripts using the same Benjamini–Yekutieli procedure applied throughout this study. Gene-set enrichment for the resulting significant transcripts was assessed with the same one-sided Fisher’s exact test and panel reference file (LBL-10779-01) described in Section 3.12, with Benjamini–Hochberg correction across the 48 functional categories and 10 Hallmark-of-Cancer themes tested, consistent with the enrichment analysis in Section 3.12.

### 2.5. Statistical Analysis

To account for varying numbers and intervals of CT examinations, PV dynamics were quantified using subject-specific longitudinal regression slopes (the PV change coefficient, mL/month), defined as the slope of a linear regression with PV as the dependent variable and age (months) as the independent variable, with negative values indicating decline. To minimize systematic tumor-related volume effects that occur only in PDAC cases near diagnosis, the primary analysis period for PDAC patients was defined as extending from the initial PV to the last CT scan obtained more than 1 year before diagnosis (Supplementary Figure S2).

This a priori truncation was based on the hypothesis that peri-diagnostic changes (obstructive pancreatitis, ductal dilation, or inclusion of tumor tissue not yet radiologically distinct from parenchyma) could transiently and artifactually increase apparent PV. Because controls lack an analogous index event, all available scans were used for controls. To confirm that this asymmetric handling did not itself generate the between-group difference, we performed a sensitivity analysis in which a comparable 12-month truncation was applied to the control group (removing each control’s final 12 months of follow-up) and, in a second variant, to both groups. Differences in PV change coefficients were compared between PDAC and control patients in both the entire cohort and the propensity-matched groups using the Mann–Whitney U test. Receiver operating characteristic (ROC) curve analysis was used to determine the optimal cutoff (Youden index) and discriminative performance. A two-sided *p* < 0.05 was considered statistically significant. Because multiple secondary and subgroup analyses (stratified by tumor location, diabetes status, pathological stage, and truncation threshold) were performed to assess the robustness of the primary finding rather than to test independent pre-specified hypotheses, no correction for multiplicity was applied to these exploratory comparisons, and their results should be interpreted accordingly. The primary analyses were conducted using JMP Pro 16 (version 16.2.0; SAS Institute Inc., Cary, NC, USA), with the methodology reviewed by a biomedical statistician. Given only 35 matched pairs, matching was restricted to age and sex to avoid over-parameterization. Propensity scores were estimated by logistic regression on age and sex; 1:1 nearest-neighbour matching without replacement (caliper 0.2 SD of the logit) yielded 35 matched pairs.

The following additional analyses were performed to assess the robustness of the primary finding.

Sensitivity analyses for confounding. Because diabetes mellitus (DM) was more prevalent in the PDAC group, the primary comparison was repeated after excluding all patients with DM, and additionally examined stratified by DM status. Because the PDAC group also had a higher prevalence of a history of other malignancies than controls, which could increase the frequency of incidental abdominal imaging, the primary comparison was additionally repeated after excluding all patients with a documented history of another malignancy (excluding the skin cancers used as the control-group eligibility criterion). A multivariable linear regression model was fitted with the PV change coefficient as the dependent variable and group, age, sex, DM status, baseline PV, and number of CT examinations as covariates.

Linear mixed-effects model. To formally account for within-patient correlation of repeated measurements and unequal observation schedules between groups, we fitted a linear mixed-effects model with PV as the dependent variable; time from each patient’s first scan (months), group, and their interaction as fixed effects, adjusted for baseline age and sex; and patient-specific random intercepts and random slopes for time [34]. Model parameters were estimated by restricted maximum likelihood (REML). The time × group interaction term is the model-based estimate of the between-group difference in the rate of PV change. Quadratic time terms were additionally tested to assess non-linearity. As an independent check on model-based standard errors, we performed a patient-level cluster bootstrap (resampling patients with replacement, 1000 replicates), refitting the model in each replicate.

**Truncation-threshold sensitivity analysis.** To evaluate whether the one-year pre-diagnostic truncation introduced bias related to disease progression, the primary analysis was repeated using no truncation and using truncation thresholds of 6 months, 2 years, and 3 years before diagnosis, using both the subject-specific slope method and the linear mixed-effects model described above.

Discriminative performance. Positive and negative likelihood ratios were calculated at the Youden-optimal cutoff. The stability of the cutoff-derived sensitivity and specificity was assessed by bootstrap resampling (2000 replicates), in which the optimal cutoff was re-derived in each bootstrap sample and applied to the original data [35]. To evaluate the incremental value of the PV change coefficient beyond routinely available clinical variables, we compared a logistic regression model containing age, sex, DM status, and baseline PV (basic covariate model) with a composite model additionally including the PV change coefficient, using the DeLong test for the difference in the area under the ROC curve (AUC) [36]; optimism-corrected AUCs were obtained by bootstrap resampling (1000 replicates). Decision curve analysis [37] was performed as an exploratory illustration of net clinical benefit; because the case–control design imposes an artificial disease prevalence, absolute net benefit values are not directly transportable to a screening population.

All additional analyses described above were implemented in Python 3.12 (numpy, scipy, scikit-learn, and statsmodels). The linear mixed-effects model was fitted by restricted maximum likelihood (REML) using the statsmodels MixedLM implementation, and its estimates were independently corroborated by the patient-level cluster bootstrap described above. No missing data were present for variables included in the primary analysis; therefore, no imputation was performed.

## 3. Results

### 3.1. Study Population Characteristics

In this study, we included 40 patients with PDAC and 61 control patients with dermatologic malignancies who underwent regular CT follow-up without pancreatic disease or chemotherapy (Figure 1). After propensity-score matching, covariate balance expressed as standardized mean differences (SMDs) is summarized in Supplementary Table S7: age and sex were well balanced (|SMD| ≈ 0), whereas the number of CT scans (SMD −0.99) and several comorbidities retained residual imbalance that is addressed by the linear mixed-effects analysis (Section 3.5). Diabetes also remained imbalanced after matching (SMD +0.33; Supplementary Table S7).

Before propensity score matching, patients with PDAC and control patients differed significantly in age (*p* = 0.014) (Table 1). However, age at the last CT scan without PDAC did not differ significantly between the two patient populations (*p* = 0.159). Regarding imaging characteristics, patients with PDAC underwent significantly fewer CT scans than control patients (9.4 ± 6.27 vs. 15.1 ± 3.83, *p* = 0.0001), reflecting the fact that control patients underwent scheduled CT surveillance whereas PDAC patients’ pre-diagnostic imaging was incidental. In contrast, the proportion of contrast-enhanced CT scans (81.9% vs. 83.1%, *p* = 0.725), observation period (92.1 ± 35.4 vs. 98.6 ± 20.7 months, *p* = 0.245), and initial PV (75.1 ± 16.5 vs. 82.2 ± 19.1 mL, *p* = 0.106) did not differ significantly between patients with PDAC and control patients. Diabetes mellitus was numerically more frequent among patients with PDAC than among control patients (15.0% [6/40] vs. 3.3% [2/61]), although this difference did not reach statistical significance (Fisher’s exact *p* = 0.055); this category included cases diagnosed prior to PDAC development as well as at the time of PDAC onset. Propensity score matching for age and sex yielded 35 pairs balanced on the matching variables; number of CT scans, which was not a matching variable, was not re-balanced by this procedure. In the matched groups, the proportions of plain and contrast-enhanced CT scans (*p* = 1.000 for both), observation period (91.5 ± 37.8 vs. 97.3 ± 21.2 months, *p* = 0.436), and initial PV (75.6 ± 16.3 vs. 78.4 ± 17.9 mL, *p* = 0.577) did not differ significantly. After matching, diabetes mellitus remained numerically more frequent in the PDAC group (11.4% [4/35] vs. 2.9% [1/35]), without statistical significance (*p* = 0.357).

### 3.2. Longitudinal Tracking of PV: Detection of Precursor Atrophy

Bland–Altman analysis showed a small mean difference in PV measurements between unenhanced and contrast-enhanced CT (mean difference 0.43 mL; SD 9.56 mL; approximate 95% limits of agreement −18.3 to 19.2 mL), indicating no substantial systematic bias between acquisition types; the limits of agreement are nonetheless wide relative to the longitudinal group difference reported below, and because a subset of the 134 paired examinations derived from the same patients, these limits should be interpreted with caution pending a repeated-measures analysis (Supplementary Figure S3). Because the primary metric is a multi-scan within-person slope, independent measurement error is averaged rather than accumulated; nevertheless, residual acquisition-related noise cannot be excluded.

**Figure 2:**
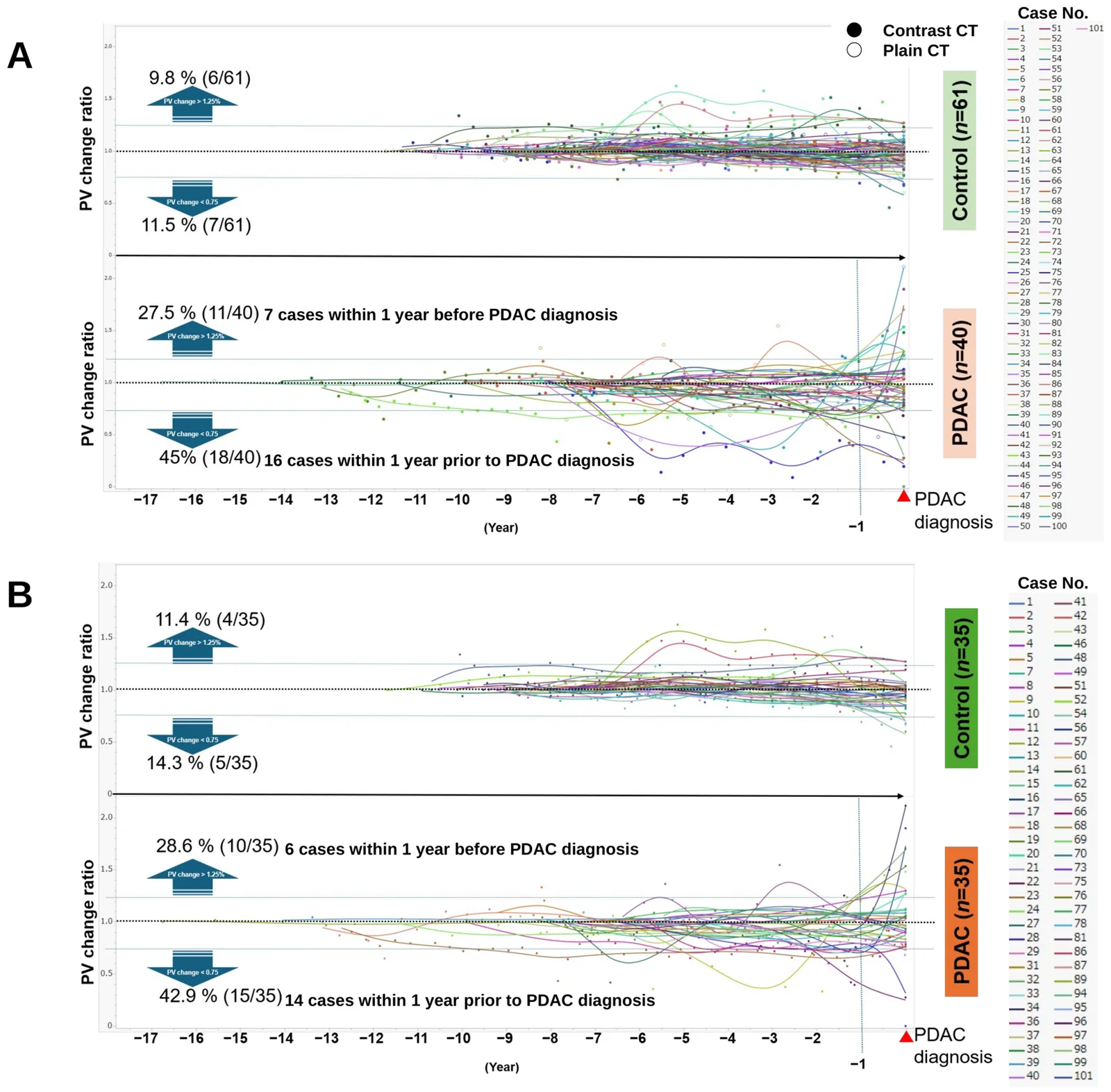
Longitudinal pancreatic volume change ratios in patients with PDAC and controls. (**A**) Full cohort. (**B**) Propensity-matched cohort.

Overall, among patients with PDAC, 27.5% (11/40) exhibited a ≥1.25-fold PV gain, while 45% (18/40) showed a ≤0.75-fold reduction, compared with 9.8% (6/61) and 11.5% (7/61), respectively, among control patients (*p* = 0.029 and *p* < 0.0001, respectively). We observed rapid PV gain within 1 year before PDAC diagnosis in 63.6% (7/11) of the patients with PDAC who exhibited PV gain. Conversely, a ≥25% PV reduction at 1 year prior to PDAC diagnosis was found in 40% (16/40) of the patients with PDAC. This percentage represented 88.9% (16/18) of the patients with PV reduction (Figure 2A). These trends were validated in the propensity-matched PDAC and control groups (≤0.75-fold PV reduction: 42.9% vs. 14.3%, *p* = 0.016) (Figure 2B).

### 3.3. Accelerated PV Reduction Rate in PDAC

**Figure 3:**
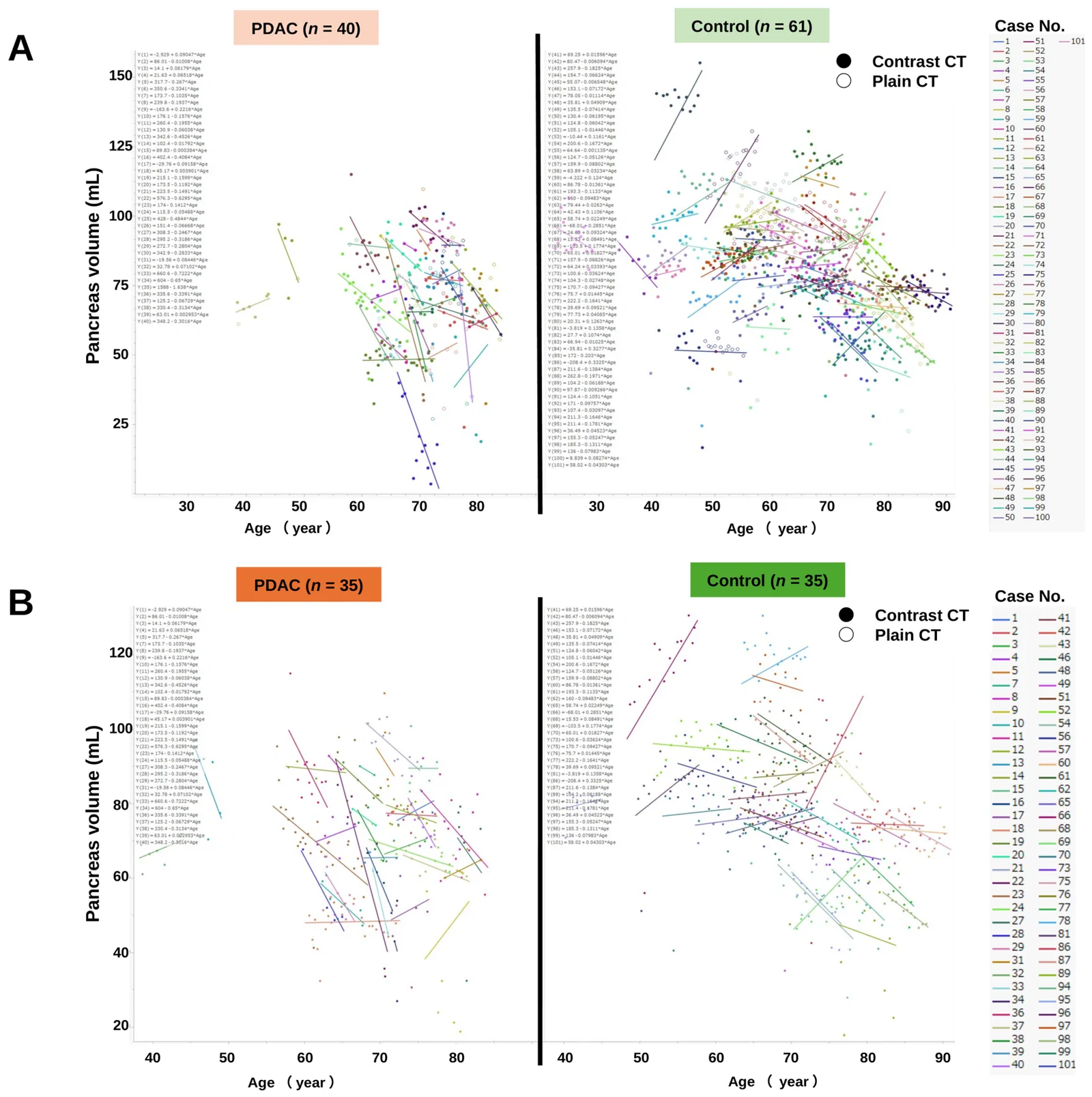
Subject-specific linear regression of pancreatic volume against age in patients with PDAC and controls. (**A**) Full cohort. (**B**) Propensity-matched cohort.

Scatter plots and simple linear regression were used to model PV as a function of age (months) using ordinary least squares regression, where the slope represented the rate of PV change. To avoid tumor-related volume contributions, only data obtained >1 year before PDAC diagnosis were analyzed and compared with those of the controls. PV decreased more rapidly in patients with PDAC than in control patients (mean PV change coefficient ± SD: −0.2119 ± 0.318 vs. −0.0068 ± 0.118, *p* < 0.0001) (Figure 3A); this finding was confirmed following propensity score matching between the PDAC and control groups (−0.1619 ± 0.2237 vs. −0.0205 ± 0.1218, *p* = 0.013) (Figure 3B). Based on the propensity-matched PV change coefficients, the estimated 5-year PV reduction was 9.71 mL in patients with PDAC and 1.23 mL in controls.

### 3.4. Sensitivity Analysis: Diabetes Mellitus and Multivariable Adjustment

Because DM was more prevalent in the PDAC group, we tested whether this confounder could account for the observed difference. After excluding all patients with DM, PDAC patients still showed significantly more rapid PV decline than controls, both in the full cohort (*n* = 34 vs. 59; mean ± SD, −0.196 ± 0.210 vs. −0.005 ± 0.118; *p* < 0.0001) and in the matched cohort (*n* = 31 vs. 34; −0.193 ± 0.217 vs. −0.016 ± 0.121; *p* = 0.00083); the effect estimate remained in the same direction and was of similar or greater magnitude than in the unrestricted analysis. A stratified analysis by DM status showed a consistent direction of effect within the small DM-positive subgroup (*n* = 6 PDAC vs. *n* = 2 controls), though this stratum was too small for formal inference.

Because the PDAC group also had a higher prevalence of prior malignancy at other sites than controls (Table 1), which could increase the frequency of incidental abdominal imaging and thereby lead to earlier detection of pre-diagnostic pancreatic changes (surveillance-related detection bias), we performed an analogous sensitivity analysis excluding all patients with a documented history of another malignancy (excluding the skin cancers used as the control-group eligibility criterion; 17 of 40 PDAC patients and 6 of 61 controls were excluded on this basis). PDAC patients still showed significantly more rapid PV decline than controls after this exclusion, both in the full cohort (*n* = 23 vs. 55; mean ± SD, −0.283 ± 0.372 vs. 0.001 ± 0.117; *p* = 1.66 × 10^−5^) and in the propensity-matched cohort (*n* = 21 vs. 30, reflecting the unbalanced numbers remaining after exclusion from the original 35-pair match; −0.218 ± 0.237 vs. −0.016 ± 0.125; *p* = 0.0020); as with the DM sensitivity analysis, the effect estimate was similarly robust, arguing against surveillance bias as the principal explanation for the observed finding.

In a multivariable linear regression model including group, age, sex, DM status, baseline PV, and number of CT examinations as covariates (*n* = 101, R^2^ = 0.236), group assignment (PDAC vs. control) remained independently associated with the PV change coefficient after adjustment (β = −0.129, 95% CI −0.234 to −0.024, *p* = 0.018), whereas DM was not independently significant in this model (β = −0.118, *p* = 0.170) (Table 2). The number of CT examinations was positively associated with the PV change coefficient (β = 0.009, *p* = 0.036). Because the number and spacing of examinations differed between groups, we additionally performed a linear mixed-effects analysis (Section 3.5) that formally accounts for the repeated-measures structure.

### 3.5. Robustness to Repeated-Measures Structure: Linear Mixed-Effects Model

**Figure 4:**
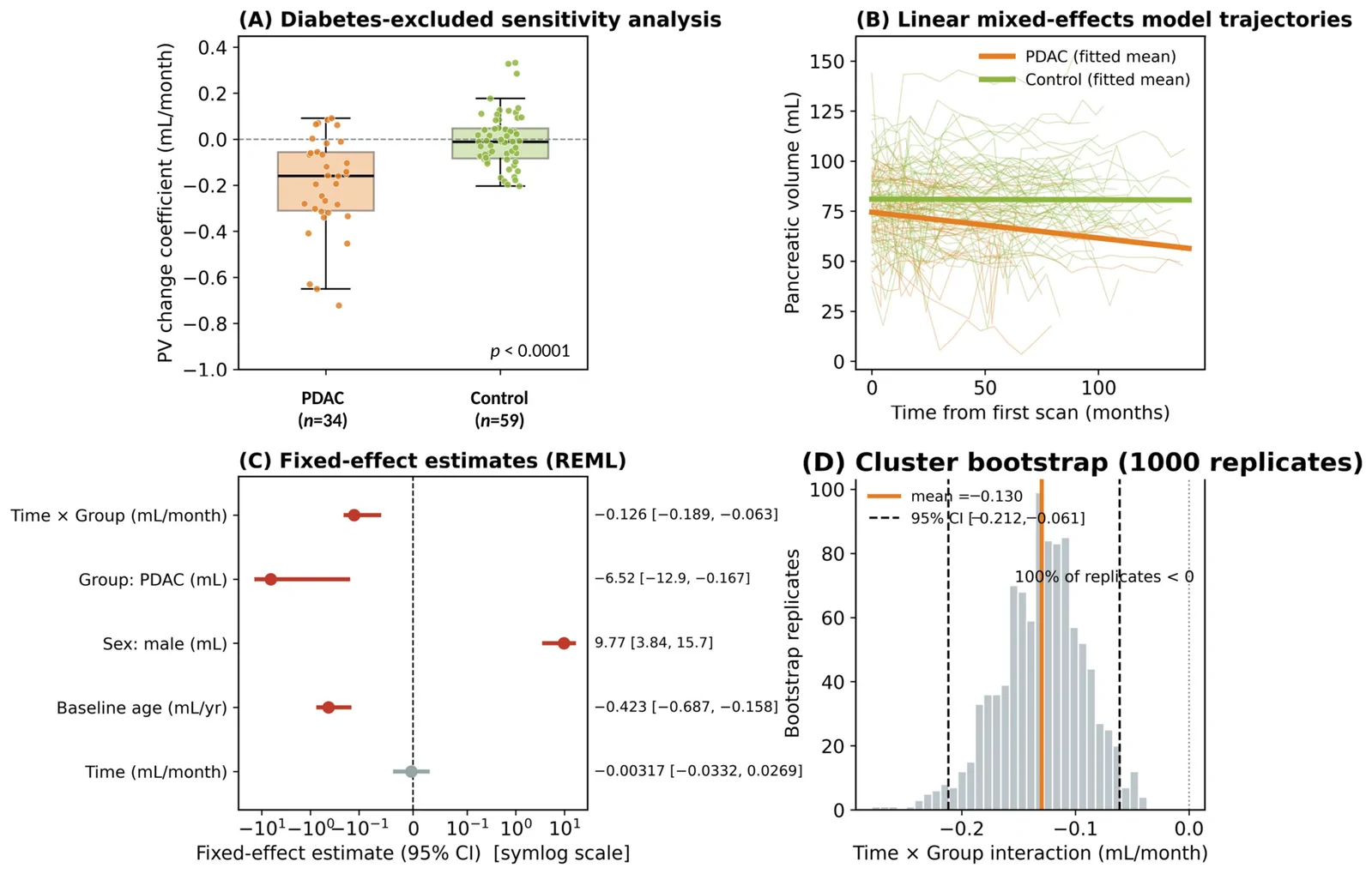
Robustness of accelerated pancreatic volume (PV) decline in PDAC to confounding and to the repeated-measures structure of the data. (**A**) Sensitivity analysis excluding patients with diabetes mellitus; boxes show median and interquartile range, whiskers extend to 1.5 × IQR, and individual patients are overlaid. (**B**) Linear mixed-effects model: individual patient trajectories (thin lines) with model-fitted group means (thick lines). (**C**) Fixed-effect estimates with 95% confidence intervals from the linear mixed-effects model (REML estimation); red indicates intervals excluding zero. (**D**) Distribution of the time × group interaction coefficient across 1000 patient-level cluster bootstrap replicates.

The time × group interaction was −0.126 mL/month (95% CI −0.189 to −0.063), indicating a significantly faster rate of decline in the PDAC group after accounting for repeated-measures correlation and unequal scan frequency (Figure 4B,C). Because PDAC patients contributed fewer scans than controls, their individual rate-of-change estimates are inherently less precise; the mixed-effects model mitigates this by borrowing strength across patients, although the reduced scan number in the PDAC group remains a source of residual uncertainty. A patient-level cluster bootstrap (1000 replicates) yielded a closely concordant estimate (mean −0.130; 95% CI −0.212 to −0.061), with 100% of replicates negative, supporting the numerical stability of this result (Figure 4D).

To assess robustness to potential non-linearity in the trajectory of PV decline, we additionally fitted an extended mixed-effects model including a quadratic time term. The time × group interaction remained highly significant (*p* = 0.00001), and an additional quadratic time × group term was also significant (*p* = 0.0043), suggesting some curvature in the trajectory of PV decline that is not fully captured by a linear approximation; this is noted as a limitation in Section 4, though it does not change the overall conclusion of accelerated decline in PDAC.

### 3.6. Sensitivity Analysis for the Pre-Diagnostic Truncation Window

Because excluding scans obtained within 1 year of diagnosis could, in principle, introduce bias related to disease progression, we repeated the analysis using the complete pre-diagnostic dataset, including scans obtained up to the day of diagnosis (376 scans in 40 PDAC patients), and using alternative truncation thresholds (Table 3).

**Figure 5:**
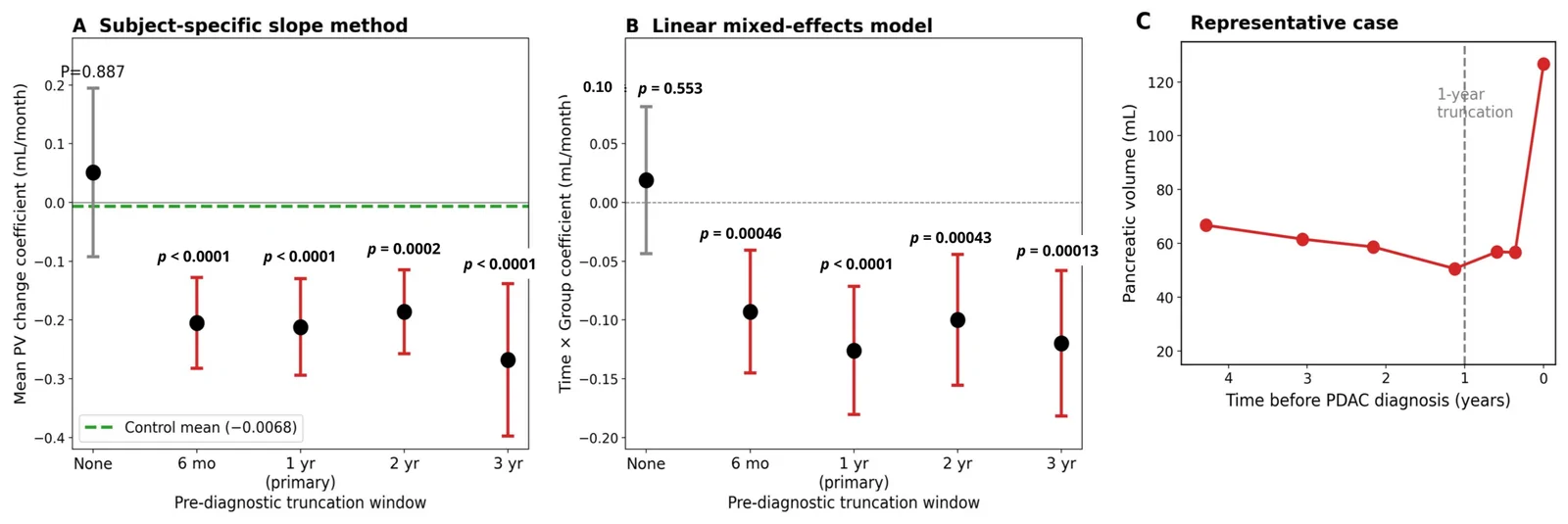
Sensitivity analysis for the pre-diagnostic truncation window. (**A**) Mean PV change coefficient in the PDAC group estimated by the subject-specific slope method at each truncation threshold, with 95% confidence intervals; the dashed green line indicates the control group mean. (**B**) Time × group interaction coefficient from the linear mixed-effects model at each threshold, with 95% confidence intervals. In both panels, red indicates *p* < 0.05 versus controls and grey indicates non-significance. (**C**) Representative case showing a transient increase in apparent pancreatic volume in the months immediately preceding diagnosis.

Without any truncation, the mean PV change coefficient in PDAC patients was positive (subject-specific slope method: +0.051; linear mixed-effects model: time × group β = +0.019) and did not differ significantly from controls (*p* = 0.89 and *p* = 0.55, respectively). Conversely, when a comparable 12-month truncation was applied to the control group, the control trajectory remained essentially flat (mean +0.016 mL/month) and the group difference persisted (PDAC −0.212 vs. control +0.016 mL/month; Mann–Whitney *p* = 9.6 × 10^−6^); applying the truncation symmetrically to both groups yielded the same conclusion (PDAC −0.201 vs. control +0.016 mL/month; *p* = 5.8 × 10^−4^), indicating that the accelerated pre-diagnostic PV loss in PDAC is not an artifact of the asymmetric truncation. From a truncation threshold of 6 months onward, the PDAC group showed a significantly and consistently more rapid PV decline than controls across all thresholds tested (6 months, 1 year, 2 years, and 3 years before diagnosis; all *p* < 0.01 by both methods) (Table 3, Figure 5). Inspection of individual trajectories identified cases in which apparent PV transiently increased in the months immediately preceding diagnosis (e.g., from 50.6 mL at 13.5 months before diagnosis to 126.7 mL on the day of diagnosis in one representative case), consistent with inclusion of tumor-related mass, peritumoral change, or obstructive ductal dilation within the segmented volume close to the time of diagnosis (Figure 5C). This indicates that the association was not highly sensitive to the choice of pre-diagnostic truncation threshold. This truncation was applied only to PDAC cases because only these patients have a tumor-related index event capable of transiently distorting apparent pancreatic volume near diagnosis; control patients, who did not develop PDAC, have no equivalent event requiring exclusion, and their full observation period was therefore retained. This asymmetric handling reflects the underlying biology rather than an arbitrary methodological choice and was not accompanied by detectable differences in age at the last CT scan without PDAC or in total observation duration between the groups (Table 1).

### 3.7. Primary Lesion Location and PV Change Coefficient

The mean PV change coefficient was significantly lower in patients with head PDAC (mean PV change coefficient ± SD: −0.2696 ± 0.400) and body-tail PDAC (−0.1646 ± 0.231) compared with that in control patients (−0.0068 ± 0.118, *p* < 0.0001 and *p* = 0.001, respectively). No significant difference was observed between the two cancer sites (*p* = 0.348) (Supplementary Figure S4A). In the matched groups, the head PDAC subgroup showed a significantly lower PV change coefficient (−0.1664 ± 0.215) compared with the control group (−0.0205 ± 0.122, *p* = 0.011). Similarly, the body-tail subgroup showed a lower value (−0.1589 ± 0.2347), but the difference was not statistically significant (*p* = 0.087) (Supplementary Figure S4B). We observed greater variability in PV change coefficients in patients with PDAC than in controls, likely reflecting both heterogeneity in background pancreatic conditions and, in part, the smaller number of scans available per PDAC patient (Section 3.4).

### 3.8. Age, Sex, and Physiological PV Decline Among Controls

Males exhibited larger PV than did females among control patients (89.0 ± 19.2 vs. 74.7 ± 15.3 mL, *p* < 0.001). Furthermore, PV declined with age, peaking in males in their 40s before decreasing; however, it gradually declined in females from their 20s (Supplementary Figure S5).

### 3.9. Discriminative Performance and Incremental Value of the PV Change Coefficient

A scatter plot of age versus the PV change coefficient (Supplementary Figure S6) demonstrated that patients with PDAC showed a wider distribution of PV change coefficients (−0.7 to 0.2) than control patients (−0.2 to 0.2).

Receiver operating characteristic (ROC) analysis yielded an area under the curve (AUC) of 0.734 (95% CI, 0.61–0.84; DeLong test, *p* = 0.001). Using an exploratory, retrospectively derived discriminative threshold of −0.135 (Youden index), sensitivity was 55.0% and specificity was 88.5%, corresponding to a positive likelihood ratio of 4.79 and a negative likelihood ratio of 0.51. This threshold requires prospective validation and is not intended as a clinically actionable cutoff at this stage. Bootstrap assessment of cutoff stability (2000 replicates) yielded modestly attenuated but consistent performance (sensitivity 51.3%, specificity 86.5%) in this cohort.

**Figure 6:**
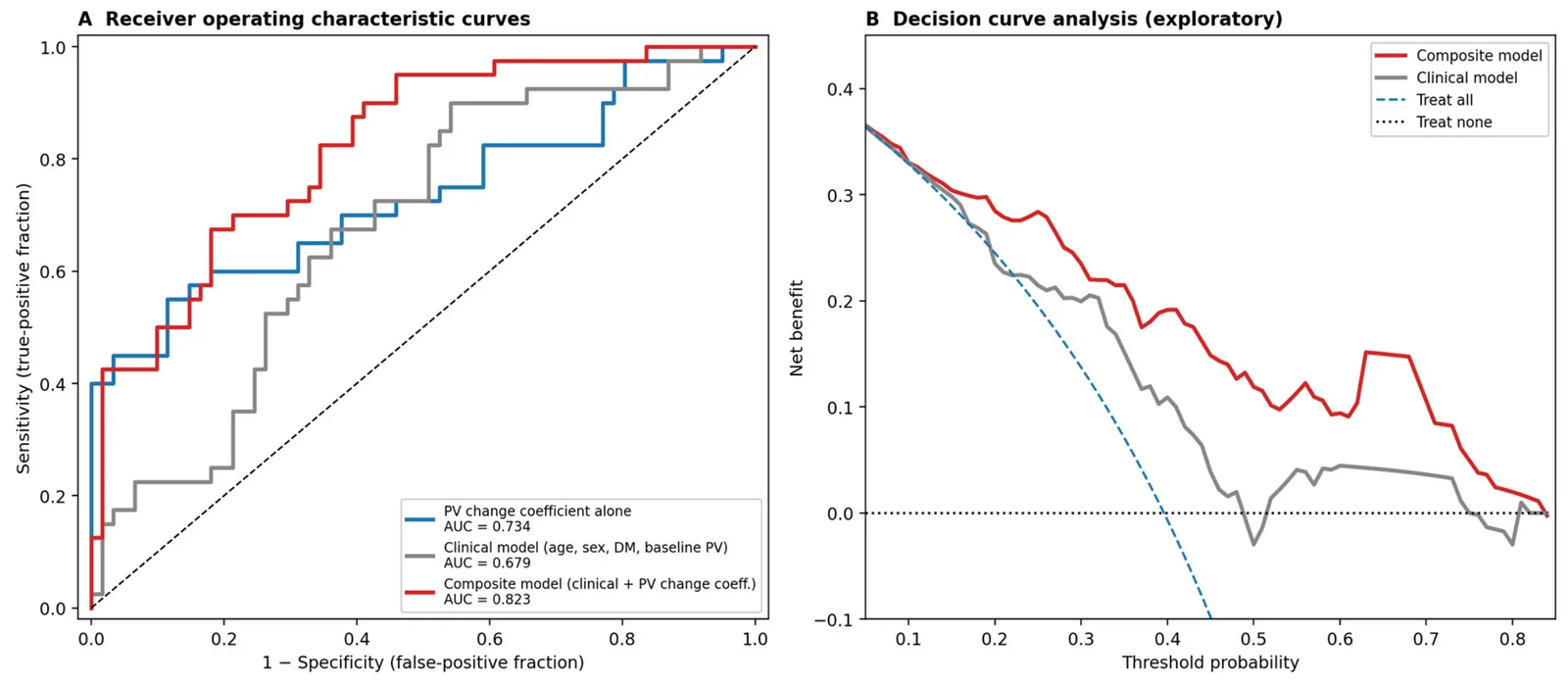
Discriminative performance and incremental value of the PV change coefficient. (**A**) Receiver operating characteristic curves for the PV change coefficient alone, a basic covariate model (age, sex, diabetes status, baseline PV), and a composite model combining both. (**B**) Exploratory decision curve analysis. Because the case–control design imposes an artificial disease prevalence, absolute net benefit values are not transportable to a screening population.

To evaluate whether the PV change coefficient provides diagnostic information beyond routinely available clinical variables, we compared a basic covariate model (age, sex, DM status, baseline PV; AUC 0.679, bootstrap-corrected 0.635) with a composite model additionally including the PV change coefficient (AUC 0.823, bootstrap-corrected 0.785). The improvement in AUC was statistically significant (ΔAUC = 0.144; DeLong Z = 3.03, *p* = 0.0024) (Figure 6A), indicating that the PV change coefficient captures information not contained in these basic clinical variables. Because the discriminative threshold (−0.135 mL/month) and the composite AUC (0.823) were derived and evaluated in the same 101-patient cohort, these apparent values are optimistic estimates of performance; the bootstrap optimism-corrected AUCs (0.635 and 0.785) are therefore reported alongside them, and external validation is required before the threshold is used as a diagnostic metric. Exploratory decision curve analysis showed that the composite model provided greater net benefit than the basic covariate model alone across a range of threshold probabilities (Figure 6B); given the artificial prevalence inherent to the case–control design, these results are presented as hypothesis-generating rather than as an estimate of net benefit in a real-world screening population.

### 3.10. Association Between Pathological Characteristics and PV Reduction

Among the nine patients who underwent surgical resection (indicated by * in Supplementary Figure S6), those with rapid PV reduction had a significantly higher prevalence of PanIN lesions (89.1% vs. 35.8%, *p* = 0.0181; Supplementary Table S1, below).

To explore whether accelerated PV loss was already detectable in early-stage disease, we stratified the 40 PDAC patients, using the pretreatment clinical UICC stage recorded in Table 1 (distinct from the pathological stage reported for the nine surgically resected cases in Supplementary Table S1; the two need not agree case-by-case, as discussed in Section 4), into early-stage (stage I–II, *n* = 19) and locally advanced or metastatic (stage III–IV, *n* = 21) groups. A declining PV trajectory was present in 63.2% of early-stage patients, compared with 90.5% of late-stage patients, and the mean PV change coefficient was numerically more negative in the late-stage group (−0.29 vs. −0.13 mL/month); however, this difference did not reach statistical significance (Mann–Whitney U test, *p* = 0.19), likely reflecting the limited number of patients within each stage stratum; the analysis was underpowered to establish a formal stage-dependent gradient (Section 4).

### 3.11. Exploratory RNA Expression Profiling in Subtypes of Pancreatic Parenchyma

**Figure 7:**
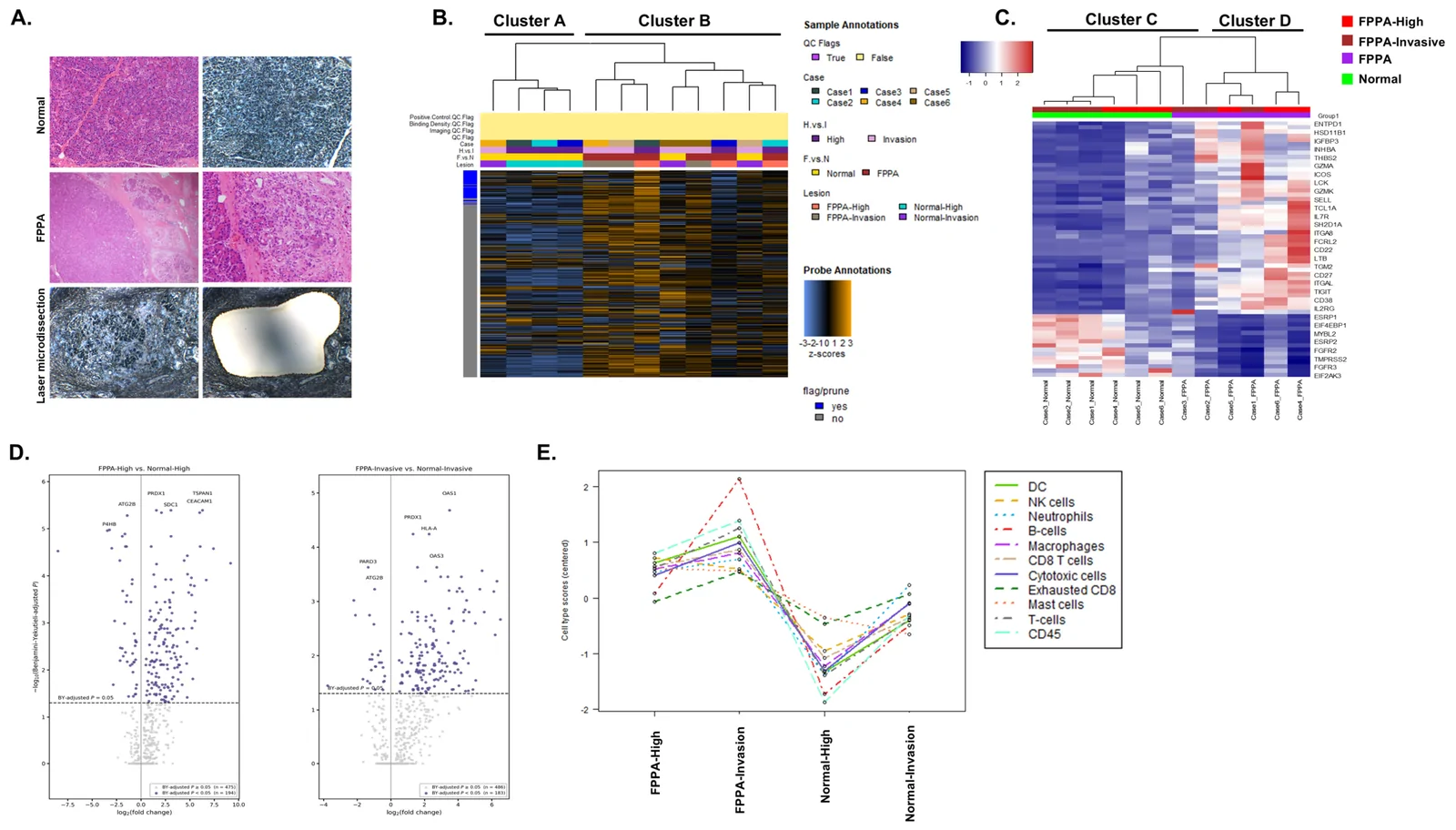
Exploratory molecular and immunological profiling of focal pancreatic parenchymal atrophy (FPPA). (**A**) Histology and laser capture microdissection. (**B**) Unsupervised hierarchical clustering of all profiled transcripts. (**C**) Clustering of differentially expressed immune- and epithelial-related transcripts. (**D**) Volcano plots for the FPPA-high-versus-normal-high and FPPA-invasive-versus-normal-invasive comparisons, plotted against Benjamini–Yekutieli-adjusted *p* values (dashed line indicates adjusted *p* = 0.05); labeled transcripts are among the most significant in each comparison (see Supplementary Figure S7 for an enlarged version with additional labeled transcripts). (**E**) Cell type scores across lesion categories.

We extracted RNA from 12 laser-microdissected acinar regions—paired atrophic (FPPA) and adjacent normal-appearing parenchyma from three patients with high-grade PanIN (FPPA-high and normal-high) and from three patients with small invasive PDAC (FPPA-invasive and normal-invasive) (Figure 7A).

Unsupervised clustering separated atrophic (FPPA) from adjacent normal-appearing parenchyma, indicating a transcriptional state distinct to FPPA rather than a uniform continuum with normal tissue: two primary clusters were identified—Cluster A, which consisted exclusively of normal tissues, and Cluster B, which included 75% of the FPPA samples (Figure 7B). Further hierarchical clustering revealed Clusters C (86% normal) and D (entirely FPPA). Cluster D exhibited increased expression of immune-related transcripts—GZMA (granzyme A), ICOS, IL7R, and TIGIT—and of the EMT-associated transcript INHBA, together with reduced expression of the epithelial markers ESRP1, ESRP2, FGFR2, and TMPRSS2 (Figure 7C). The direction of change for all nine transcripts was concordant with the formal differential expression analysis in both the FPPA-high and FPPA-invasive comparisons. Three transcripts reached the Benjamini–Yekutieli-adjusted significance threshold in both comparisons (ICOS, adjusted *p* = 0.0013 and 0.0016; IL7R, *p* = 0.0077 and 0.0141; TIGIT, *p* = 0.0414 and 0.0140, in the FPPA-high and FPPA-invasive comparisons, respectively); GZMA reached significance in the FPPA-high comparison only (*p* = 0.0143; *p* = 0.068 in FPPA-invasive), and ESRP1 in the FPPA-invasive comparison only (*p* = 0.0295; *p* = 1.0 in FPPA-high); TMPRSS2, INHBA, ESRP2, and FGFR2 did not reach significance in either comparison (all *p* ≥ 0.05), and these six transcripts without concordant significance in both comparisons should therefore be regarded as descriptive features of the clustering rather than as statistically supported differences.

Differential expression analysis with BY-adjusted *p* values showed upregulation of adhesion- and EMT-associated transcripts in both comparisons, including ITGA8 (linear fold change 8.36, BY-adjusted *p* = 0.0009, in the FPPA-high comparison; 6.00, *p* = 0.0068, in the FPPA-invasive comparison) and ZEB1 (2.83, *p* = 0.0195, and 3.30, *p* = 0.0100, respectively), together with immune- and metabolism-related transcripts. Fold changes for KRT17 were higher in the FPPA-invasive than the FPPA-high comparison (91.0, 95% CI 19.5–424.0 vs. 52.3, 95% CI 11.2–244.0), consistent with greater epithelial-marker induction in the invasion-adjacent lesion group. Fold changes, 95% confidence intervals, and BY-adjusted *p* values for the transcripts significant in both comparisons are summarized in Supplementary Table S3.

To address the inherent statistical fragility of differential expression analysis based on three samples per group, we applied the conservative Benjamini–Yekutieli procedure to control the false discovery rate. Despite the small sample size, 27.4–29.0% of the 669 profiled transcripts remained significant after correction in each of the two comparisons (FPPA-high vs. normal-high: 194/669; FPPA-invasive vs. normal-invasive: 183/669; both BY *p* < 0.05). To assess whether this reflected a consistent biological signal rather than small-sample noise, we examined the overlap between the two comparisons: 131 genes were significantly and concordantly differentially expressed (BY *p* < 0.05) in both comparisons, including T-cell- and interferon-related transcripts (CD3D, CD3E, CD3G, CD4, LCK, IL2RG, IL7, HLA-A, B2M, OAS1, OAS2, OAS3) and cell-cycle-related transcripts (CDK1, BUB1, TOP2A, TPX2, UBE2C, MKI67, FOXM1), consistent with the pathway-level findings described in Section 3.12. All 131 genes were concordant in direction of fold change between the two comparisons (Supplementary Table S3).

The 131 genes significant in both the FPPA-high and FPPA-invasive comparisons were, by construction, concordant in direction of change between the two comparisons; all 131 (100%) showed the same sign of log2 fold change in both comparisons (Supplementary Table S3). Functional annotation of these genes, together with the FPPA-high and FPPA-invasive significant gene sets individually, against the nCounter Tumor Signaling 360 panel’s built-in gene-set categories (48 functional categories and 10 Hallmark-of-Cancer themes) was re-derived using the panel’s category-membership reference file (LBL-10779-01) and is reported in full in Supplementary Table S4 and summarized in Section 3.12. Transcripts with the largest fold changes showed correspondingly wide confidence intervals; for example, SERPINB5 in the FPPA-high comparison had a linear fold change of 577 with a 95% CI of 116–2860. Such intervals span more than an order of magnitude and reflect the limited precision obtainable from three samples per group. These estimates should therefore be interpreted as indicating direction and approximate magnitude rather than as precise quantification.

### 3.12. Pathway Enrichment and Immune Profiling in FPPA

Pathway enrichment analysis was re-derived for the corrected FPPA-high/FPPA-invasive comparisons described in Section 2.4, using the nCounter Tumor Signaling 360 panel’s gene-set annotation reference file (LBL-10779-01; 48 functional categories and 10 Hallmark-of-Cancer themes) and one-sided Fisher’s exact tests with Benjamini–Hochberg correction applied within each comparison (Supplementary Table S4). The FPPA-invasive vs. normal-invasive comparison showed significant enrichment, after correction, of several immune-related categories, most prominently the Avoiding Immune Destruction Hallmark theme (49 of 97 panel genes, BH-adjusted *p* = 0.000001) and the Antigen Presentation functional category (12 of 15 panel genes, BH-adjusted *p* = 0.0013), together with T-cell Costimulation, TCR Signaling, and Interferon Response (BH-adjusted *p* = 0.012, 0.026, and 0.026, respectively). The FPPA-high vs. normal-high comparison showed only nominal enrichment (raw *p* < 0.05) of several categories, including Antigen Presentation and DNA Damage Repair, none of which remained significant after correction for multiple comparisons. Among the 131 transcripts concordant across both comparisons (Section 3.11), the Avoiding Immune Destruction theme (BH-adjusted *p* = 0.0049) and Antigen Presentation category (BH-adjusted *p* = 0.027) remained significantly enriched, consistent with a predominantly immune-related signature, with several immune-related changes showing greater magnitude in the invasive-PDAC-adjacent comparison than in the high-grade-PanIN-adjacent comparison. Regarding immune cell type scores, B cells and macrophages remained relatively stable in the normal-high area; however, they were increased in the normal-invasive area, with further elevation observed in the FPPA-high and FPPA-invasive areas. We observed a similar trend for other inflammatory cell types. However, no significant differences were detected among the regions of exhausted CD8+ T and mast cells (Figure 7E).

### 3.13. Interferon and Antigen-Presentation Transcripts Show Numerically Larger Fold Changes in the FPPA-Invasive Comparison

Among the 131 transcripts significantly and concordantly altered in both the FPPA-high and FPPA-invasive comparisons (Section 3.11, Supplementary Table S3), six transcripts encoding components of the type I interferon response and MHC class I antigen-presentation machinery showed a consistently larger fold change in the FPPA-invasive than the FPPA-high comparison: OAS1 (linear fold change 8.43 vs. 11.3), OAS2 (7.69 vs. 10.5), OAS3 (5.76 vs. 6.65), HLA-A (2.82 vs. 4.81), B2M (2.19 vs. 3.04), and IL7 (5.56 vs. 5.74); all remained Benjamini–Yekutieli-adjusted significant (*p* < 0.05) in both comparisons (Supplementary Table S3). Because the corrected sample scheme (Section 2.4) retains only two lesion categories, this pattern should be interpreted as a directional difference between the FPPA-high and FPPA-invasive comparisons rather than as evidence of a graded, multi-stage dose–response relationship; establishing a true progression gradient would require differential expression data from additional, histologically intermediate lesion categories that are not part of the corrected sampling design.

### 3.14. Transcriptomic Differences Between Microdissected Precursor PanIN and Invasive Carcinoma Lesion Regions

**Figure 8:**
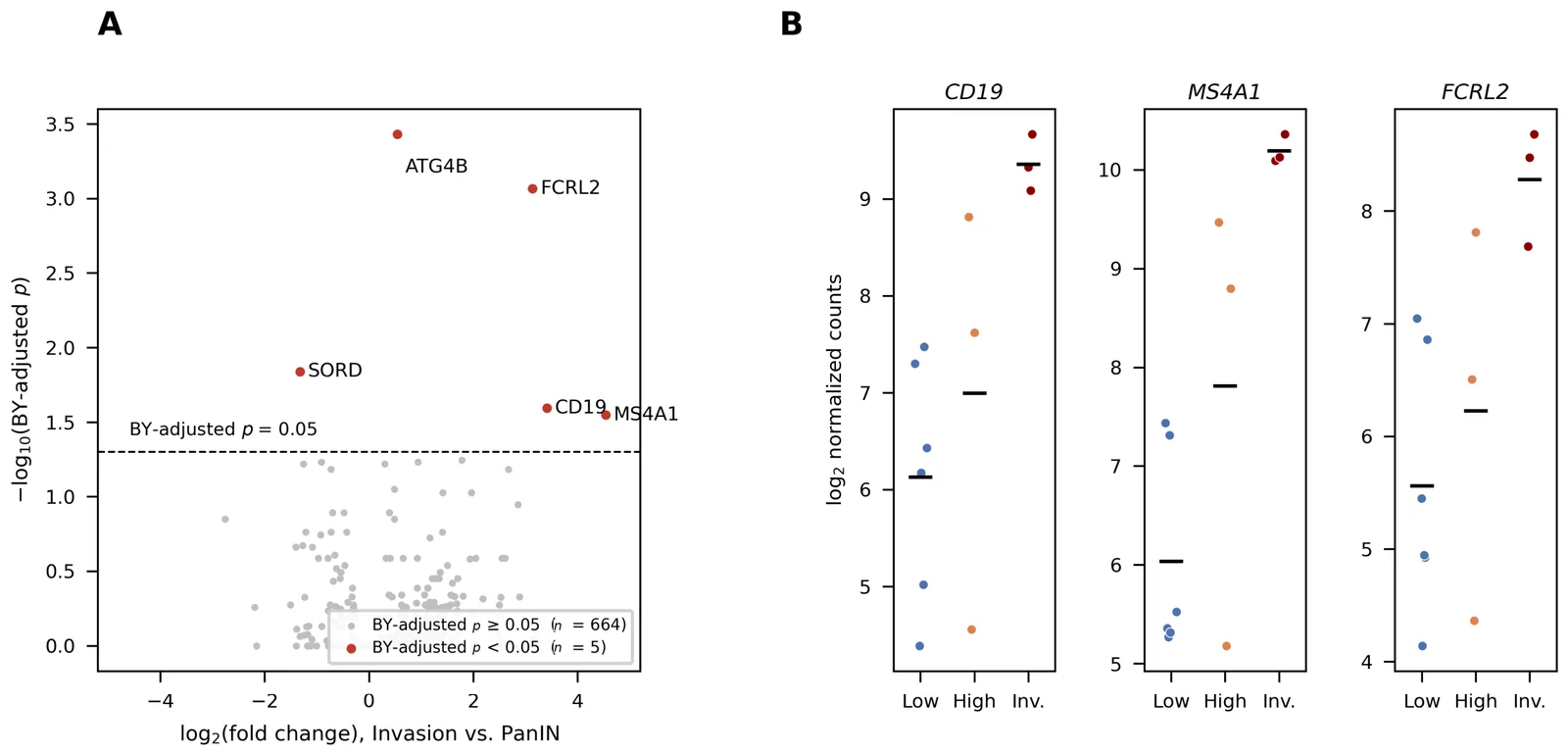
Epithelial transcriptomic comparison between precursor PanIN and invasive carcinoma regions. (**A**) Volcano plot for the pooled PanIN-epithelium (low-grade and high-grade) versus invasive-epithelium comparison, plotted against Benjamini–Yekutieli-adjusted *p* values from the patient-level mixed-effects model (dashed line indicates adjusted *p* = 0.05); the five significant transcripts are labeled. (**B**) Expression (log2 normalized counts) of the three B-cell Function transcripts driving the enrichment result (CD19, MS4A1, FCRL2) across low-grade PanIN, high-grade PanIN, and invasive epithelium regions; horizontal bars indicate group means. Each point represents an individual sample, and point colour indicates lesion category (blue, low-grade PanIN; orange, high-grade PanIN; dark red, invasion), corresponding to the groups shown on the x-axis (Low, High, Inv.); horizontal bars denote the group median.

To test whether a precursor-to-invasive transcriptomic transition could be detected within the microdissected epithelial-lesion compartment, using the additional epithelial regions described in Section 2.4, we compared the pooled PanIN-epithelium group (low-grade and high-grade PanIN epithelium, nine regions from all six patients) against the invasive PDAC-epithelium group (three regions, from the three small-invasive-PDAC patients), using a linear mixed-effects model with a patient-level random intercept to account for within-patient correlation among repeated epithelial regions (Figure 8A). Five of the 669 tested transcripts reached Benjamini–Yekutieli-adjusted significance (BY *p* < 0.05): ATG4B (1.46-fold higher in invasive than PanIN epithelium, 95% CI 1.38–1.54, BY *p* = 0.00037), FCRL2 (8.79-fold higher, 95% CI 6.11–12.65, BY *p* = 0.00086), SORD (2.50-fold higher in PanIN than invasive epithelium, 95% CI 2.01–3.11, BY *p* = 0.0145), CD19 (10.65-fold higher in invasive epithelium, 95% CI 5.72–19.83, BY *p* = 0.0255), and MS4A1/CD20 (23.19-fold higher, 95% CI 9.83–54.71, BY *p* = 0.0283). Gene-set enrichment analysis of these five transcripts against the panel’s 48 functional categories and 10 Hallmark-of-Cancer themes (LBL-10779-01) identified a single category that remained significant after Benjamini–Hochberg correction: B-cell Function, in which three of the five B-cell Function genes represented among the 669 tested transcripts (CD19, MS4A1, and FCRL2) were among the five significant transcripts (odds ratio 496.5, raw *p* = 0.000002, Benjamini–Hochberg-adjusted *p* = 0.000096). Expression of all three B-cell-associated transcripts increased in a step-wise manner from low-grade PanIN, through high-grade PanIN, to invasive epithelium (Figure 8B), a pattern consistent across all six patients despite the very small per-group sample size.

## 4. Discussion

The principal contribution of this study is not that pancreatic volume was lower in patients who later developed PDAC, but that longitudinal, within-person volume trajectories differed before diagnosis and evolved over time. Four analytically distinct approaches—subject-specific regression slopes, multivariable adjustment, a linear mixed-effects model, and a sweep of pre-diagnostic truncation thresholds—converged on the same direction of effect, and the marker retained incremental discriminative value beyond routinely available clinical variables. Throughout, we distinguish three levels of certainty: what the data directly demonstrate (a difference in the rate of pancreatic volume change between patients who later developed PDAC and comparably surveilled controls, robust to the analytic method used); what is supported but not proven (that this reflects a biologically coherent, evolving pre-neoplastic process rather than unrelated statistical artifacts); and what remains hypothesis (the tissue-level mechanism and any clinical utility). Consistent with its introduction as a pilot investigation, the tissue-level and molecular material discussed below is secondary and hypothesis-generating in scope; the imaging finding stands independently of it and does not depend on any of these tissue-level interpretations being correct.

Because it is the pattern across analyses, not any single *p* value, that is informative, four separate manipulations each removed a candidate alternative explanation without abolishing the signal: restricting to age- and sex-matched pairs left the difference intact; excluding all patients with diabetes strengthened rather than weakened it [38,39,40]; excluding patients with any prior non-skin malignancy—whose additional cancer surveillance could have led to earlier incidental detection—preserved a difference of similar or greater magnitude; and a mixed-effects model built for the unequal number and spacing of scans reproduced the same estimate, with a patient-level bootstrap confirming its stability. Exact effect sizes, confidence intervals, and *p* values are reported in the corresponding Results subsections. Had any of these been the dominant explanation, removing it should have weakened, not preserved, the signal.

These analyses cannot, however, eliminate every alternative explanation. The controls remain a surveilled cancer cohort rather than a disease-free reference population and may differ from PDAC patients in ways the covariates did not capture (systemic inflammatory burden, metabolic status, body composition, smoking). CT acquisition was heterogeneous by clinical necessity, mixing contrast-enhanced and unenhanced examinations; although we found no substantial group-level mean bias between acquisition types, this does not guarantee equivalent per-scan precision. Scan density remained unequal even after matching, and while the mixed-effects model tolerates this, it rests on assumptions—a single random-slope structure and approximate normality of random effects—that are difficult to verify definitively at this sample size. We regard these as open, not resolved, sources of uncertainty. The between-group imbalance in scan frequency persisted after matching and constitutes a structural asymmetry; although the mixed-effects framework mitigates its impact, residual influence on precision cannot be fully excluded.

The behavior of the signal over time is more informative than its mere presence. Restricted to scans obtained more than one year before diagnosis, PDAC patients showed consistently more negative slopes than controls across truncation thresholds from 6 months to 3 years; with no truncation the group difference vanished and the point estimate even reversed sign (Section 3.6), reflecting a transient increase in apparent volume in a subset of patients immediately before diagnosis (Section 3.2). Together with the significant quadratic time × group term (*p* = 0.0043, alongside a linear term that remained highly significant, *p* = 0.00001), these data are compatible with a two-phase trajectory: a gradual, longer-term loss of volume dominating from more than a year out, and a late, non-linear inflection in which apparent volume rises as diagnosis approaches. The long-term decline phase and its disappearance without truncation are directly demonstrated; the specific decline-then-rise shape is supported but not proven, since the quadratic term shows only that a straight line is inadequate, not which process produces the curvature. Candidate explanations for the late rise—incorporation of tumor tissue not yet radiologically distinct from parenchyma, obstructive ductal dilation, or peritumoral inflammatory or desmoplastic change—remain hypotheses, as no histological or radiological adjudication was performed. Practically, the marker is informative from roughly a year or more before diagnosis and uninformative or misleading in the final months. This temporal structure also explains why a within-person slope, rather than an absolute-volume threshold, is the defensible unit of analysis: absolute volume varies with sex, age, and body habitus [41,42,43,44]—variation our control data reproduced (Section 3.8)—whereas a slope uses each patient as their own reference and can reveal both phases that a single cross-sectional measurement cannot.

Equally informative is that the association was not universal: roughly 45% of PDAC patients showed no rapid pre-diagnostic volume loss, and its magnitude varied widely. This heterogeneity is compatible with genomic evidence that PDAC does not follow a single evolutionary route [45,46]: if only a subset arises through a route in which a slowly evolving, field-level parenchymal process precedes invasion, a volumetric marker would by construction flag that subset and miss the rest regardless of measurement precision. This is consistent with the marker’s sensitivity ceiling and with a nonsignificant stage gradient in the expected direction—a decline in PV trajectory present in 63.2% of early-stage versus 90.5% of late-stage patients (Section 3.10)—that is compatible with pancreatic volume decline beginning before locally advanced or metastatic spread, in line with recent reports linking atrophy patterns to intraductal extension specifically in early-stage disease and describing a shorter interval between atrophy recognition and diagnosis among surgically resected patients [24,25]. We frame this as a plausible explanation, not as evidence of distinct molecular subtypes, which we did not test. It follows that a negative trajectory does not argue against PDAC arising by another route, and the marker should not be read as excluding disease when absent.

These considerations shape how the marker’s discriminative performance should be read. Its high-specificity, low-sensitivity profile (Section 3.9) shifts probability substantially when positive but does little to reassure when negative—the profile of a risk-enrichment signal suited to raising suspicion in a patient who already has other reasons for concern, not a sensitive test for excluding disease or a population screen. Adding the coefficient to a basic clinical model (age, sex, diabetes, baseline volume) improved discrimination from an AUC of 0.679 to 0.823, indicating information orthogonal to those variables and hence value as one input in a multivariable risk framework rather than in isolation. Because the cutoff was derived and evaluated in the same case–control sample, in which prevalence is fixed by design far above any real surveillance population, these operating characteristics describe internal consistency, not performance that transfers directly to screening or high-risk settings.

Whether this volumetric phenotype has a tissue-level counterpart we address as a hierarchy of decreasing certainty. Among the nine surgically resected cases, PanIN-positive slide prevalence was higher in the six patients with rapid volume loss than in the three without (89.1% vs. 35.8%, *p* = 0.0181); with nine patients, a same-cohort cutoff, and no adjustment for tumor location, size, or sampling density, this provides histopathological plausibility for a link between rapid volume loss and precursor-lesion burden, not evidence that PanIN causes acinar atrophy or of any direction of effect—a shared upstream process could produce both. A structural precedent for how such a link could plausibly arise at the whole-organ level comes from 3D genomic mapping showing that grossly normal human pancreas harbors a mean of 13 spatially separate PanINs per cm^3^, extrapolating to hundreds of precursor lesions per organ [47]; a comparably distributed field of precursor lesions, rather than a single focal event, offers one structural explanation for how precursor-lesion burden could aggregate into a measurable, whole-organ volumetric signal, although this inference is drawn from a separate cohort and imaging modality and was not tested directly in the present study. Transcriptomically, we profiled two distinct, non-overlapping tissue compartments from six patients: laser-microdissected atrophic acinar regions (FPPA) versus adjacent normal-appearing acinar parenchyma (12 regions; two three-versus-three comparisons), and, separately, neoplastic epithelium across low-grade PanIN, high-grade PanIN, and invasive carcinoma (12 additional regions; 24 in total). Within the microdissected FPPA regions—which sampled atrophic acinar parenchyma together with its local microenvironment of immune cells, fibroblasts, and residual epithelium, rather than a purified acinar-cell fraction—131 of 669 tested transcripts were concordantly altered in both the FPPA-high and FPPA-invasive comparisons after conservative correction. These changes included T-cell-, interferon-response-, and cell-cycle-related transcripts, while ITGA8 and ZEB1 were significantly upregulated in both comparisons and selected epithelial markers were reduced in the clustering analysis. Thus, rather than a passive end-stage of acinar dropout, FPPA appears to be a biologically active local parenchymal state—transcriptionally distinct from immediately adjacent normal-appearing acinar tissue and characterized by immune engagement, altered adhesion and cell-plasticity programs, and tissue remodeling—that is present even where the neighboring epithelial lesion is a non-invasive high-grade PanIN. However, because formal pathway enrichment predominantly supported immune-related programs rather than an independently enriched EMT program, the EMT-associated findings are more appropriately interpreted as transcriptional features compatible with acinar-parenchymal remodeling rather than as evidence of a definitive EMT signature.

The temporal and causal relationship between the two compartments remains unresolved. Progressive remodeling and loss of the FPPA parenchymal compartment is one plausible tissue-level process that could contribute to the macroscopic, longitudinal pancreatic volume loss detectable on CT before PDAC diagnosis. This plausibility is bolstered by recent spatial multi-omic mapping showing that the PanIN-adjacent microenvironment already diverges transcriptionally from normal parenchyma, with an inflammatory and epithelial–mesenchymal transition-associated pattern that shifts further as lesions progress toward invasion [48]; however, an independent donor-pancreas spatial atlas found that the epithelial and microenvironmental compartments evolve asynchronously rather than in lockstep, with the precursor-lesion microenvironment differing qualitatively, and not merely in degree, from that of established cancer [49]. Our two-compartment interpretation is therefore consistent with an actively evolving peri-lesional parenchyma, but the stronger claim that this remodeling is quantitatively ‘the microscopic counterpart’ of the imaging signal is not established by the present data: the longitudinal imaging cohort (*n*  =  101) and the microdissected-tissue cohort (*n*  =  6) only partially overlapped (the three small-invasive-PDAC tissue cases were drawn from the imaging cohort, whereas the three high-grade-PanIN cases were additional patients), with no patient-level analysis linking longitudinal PV trajectories to transcriptomic profiles, so the imaging and molecular observations are linked only at the level of the disease process rather than by direct within-individual correspondence, and this linkage should be treated as an unproven hypothesis pending studies that pair longitudinal imaging with tissue sampling in the same patients.

The separate epithelial analysis provides a complementary perspective. Five transcripts differed between pooled PanIN and invasive epithelium, and three B-cell Function transcripts (CD19, MS4A1, and FCRL2) increased step-wise from low-grade PanIN through high-grade PanIN to invasive epithelium. Because the present bulk targeted-transcript assay on microdissected regions cannot determine whether these transcripts arose from the neoplastic epithelial cells themselves or from tightly associated immune cells, this finding should be interpreted as a lesion-associated rather than an epithelial-cell-specific signal. It nonetheless changed in a directionally concordant manner with the directionally greater interferon- and antigen-presentation-related signal in the adjacent FPPA parenchymal compartment described above (Section 3.13), across the PanIN-to-invasion interval, though the two were not analyzed as a single mechanistically linked pathway. Taken together, the epithelial-lesion regions and FPPA parenchymal regions each showed molecular differences associated with progression from precursor disease toward invasion, supporting an exploratory two-compartment model without establishing direct mechanistic coupling between them, and potentially reflecting bidirectional epithelial–parenchymal signalling [50,51,52,53,54,55,56]. This interpretation remains exploratory: each molecular comparison rests on three to six purposively selected patients, the paired lesion–normal structure of the acinar-region sampling was not modeled as such, and no independent or spatially resolved replication exists. Accordingly, these observations are compatible with, but do not prove, an evolving pre-neoplastic field in which acinar remodeling accompanies PanIN progression and may underlie the radiological atrophy signal.

In principle, because segmentation is fully automated and comparable across contrast-enhanced and unenhanced CT, pancreatic volume could be computed opportunistically from examinations performed for unrelated indications and accumulated within a picture archiving and communication system [57], feeding existing risk-assessment frameworks [58,59] or escalation pathways such as those defined by the CAPS consortium [60]. This is a direction for prospective evaluation, not something tested here, and is constrained by the years-long interval a stable slope requires and by the marker’s loss of information near diagnosis.

The between-group imbalance in scan frequency, which persisted after matching but was not accompanied by imbalance in age at last scan or observation duration, remains a structural asymmetry. Residual confounding from variables we could not retrieve—body-mass index, smoking, quantitative pancreatic fat fraction [61,62], and diabetes duration—cannot be excluded. In particular, height, body weight and longitudinal weight change (and therefore body-mass index), together with smoking history, were not reliably documented in the medical records and could not be examined, although the within-person slope design reduces sensitivity to stable between-person differences in body composition. No external validation cohort exists, and the discriminative cutoff was derived and tested within the same sample. Finally, the histopathological (*n* = 9) and transcriptomic (*n* = 3–6 per comparison) analyses are small and exploratory. Moreover, because these transcriptomic analyses used bulk RNA from microdissected regions containing a mixture of epithelial, immune, and stromal cells, they cannot attribute expression changes to a specific cell type, and the immune- and remodeling-related signals may derive substantially from infiltrating non-epithelial cells; single-cell or spatial transcriptomic approaches would be required for cell-type resolution. Accordingly, secondary and subgroup analyses are underpowered and should be regarded as hypothesis-generating; the exploratory transcriptomic substudy (three to six regions per group) is not confirmatory.

We therefore regard the longitudinal volumetric association as the most secure finding, its two-phase temporal structure as well-supported but not mechanistically explained, and its histopathological and molecular correlates—along with any clinical application—as hypotheses for a prospective, multicenter study with standardized imaging protocols, a realistic target population, and pre-specified sampling.

## 5. Conclusions

In this retrospective single-center case–control cohort, accelerated pancreatic volume loss was detectable on routine CT more than one year before PDAC diagnosis and was robust to adjustment for diabetes and other measured confounders, to formal modelling of the repeated-measures structure of the data, and to the choice of pre-diagnostic truncation window. The marker adds information beyond routinely available clinical variables, but its 55.0% sensitivity precludes stand-alone screening; its high-specificity profile instead suits a risk-enrichment role within existing surveillance frameworks. Exploratory transcriptomic profiling showed that FPPA—including where the adjacent lesion was a non-invasive high-grade PanIN—was biologically distinct from immediately adjacent normal-appearing acinar parenchyma, with concordant immune-related changes and additional transcriptional features compatible with acinar-parenchymal remodeling, indicating an actively remodeling rather than passively atrophic parenchymal state already present before invasion. These findings raise the hypothesis that remodeling and loss of the FPPA parenchymal compartment accompany pre-invasive PanIN progression—consistent with a two-compartment process in which the peri-lesional acinar parenchyma evolves in parallel with the neoplastic epithelium rather than as passive atrophy secondary to established invasive cancer—and may provide a tissue-level substrate for FPPA and for the longitudinal pancreatic volume loss detectable on CT. Importantly, because the imaging and tissue analyses were performed in only partly overlapping patients, with no patient-level imaging–tissue correlation, these tissue findings do not establish that parenchymal remodeling explains the whole-organ volume loss observed on CT; this biological link remains an untested hypothesis until future studies examine serial imaging and tissue from the same individuals. Because measurement is fully automated and showed no substantial systematic bias between unenhanced and contrast-enhanced acquisitions, serial pancreatic volume could in principle be accumulated opportunistically from imaging already performed for unrelated indications. In summary, accelerated pre-diagnostic pancreatic volume loss is a reproducible, analytically robust signal that may enrich populations for pancreatic-cancer surveillance. Its single-center, retrospective derivation and the absence of external validation are the principal limitations; prospective, multi-center studies that pair serial imaging with same-patient tissue analysis are the essential next step toward translating this marker into practice.

## Figures and Tables

**Table 1:** Baseline Characteristics of Patients.

Characteristic	All Patients (*n* = 101)	PDAC (*n* = 40)	Control (*n* = 61)	*p* Value	PDAC, Matched (*n* = 35)	Control, Matched (*n* = 35)	*p* Value (Matched)
Age at last CT scan, years, median (IQR)	72 (63–78.5)	74.5 (71–79)	71 (58.5–77.5)	0.014	73 (71–79)	75 (71–81)	0.787
Age at last PDAC-free CT scan †, years, median (IQR)	71 (63–77)	72 (67.5–77)	71 (58.5–77.5)	0.159	68 (62–71)	66 (61–75)	0.972
Sex, male, *n* (%)	57 (56.4%)	24 (60.0%)	33 (54.1%)	0.558	20 (57.1%)	20 (57.1%)	1.000
CT examinations, total	1295	376	919	—	331	509	—
Number of CT scans, mean ± SD	12.8 ± 5.65	9.4 ± 6.27	15.1 ± 3.83	0.0001	9.5 ± 6.16	14.5 ± 3.6	0.0001
Unenhanced CT, *n* (%)	223 (17.2%)	68 (18.1%)	155 (16.9%)	0.264	66 (19.9%)	102 (20.0%)	1.000
Contrast-enhanced CT, *n* (%)	1072 (82.8%)	308 (81.9%)	764 (83.1%)	0.725	265 (80.1%)	407 (80.0%)	1.000
Observation period, months, mean ± SD	96.0 ± 27.5	92.1 ± 35.4	98.6 ± 20.7	0.245	91.5 ± 37.8	97.3 ± 21.2	0.436
Tumor location, Head:Body-tail, *n*	—	18 (45%):22 (55%)	N.A.	—	14 (40%):21 (60%)	—	—
Maximum tumor diameter, mm, mean ± SD	—	30.4 ± 10.0	N.A.	—	31.9 ± 8.7	—	N.A.
Initial pancreatic volume, mL, mean ± SD	79.4 ± 18.3	75.1 ± 16.5	82.2 ± 19.1	0.106	75.6 ± 16.3	78.4 ± 17.9	0.577
Clinical stage (UICC 8th), IA/IB/IIA/IIB/III/IV, *n*	—	2/11/5/1/11/10	N.A.	—	1/10/4/1/11/8	—	N.A.
Dermatologic malignancy, *n* (%)	61 (60.4%)	0	61 (100%)	—	0	35 (100%)	—
Diabetes mellitus, *n* (%)	8 (7.9%)	6 (15.0%)	2 (3.3%)	0.055	4 (11.4%)	1 (2.9%)	0.357
Colon cancer, *n* (%)	9 (8.9%)	8 (20%)	1 (1.6%)	0.024	6 (17.1%)	1 (2.9%)	0.106
Breast cancer, *n* (%)	8 (7.9%)	5 (12.5%)	3 (4.9%)	0.259	1 (2.9%)	3 (8.6%)	0.614
Lung cancer, *n* (%)	7 (6.9%)	6 (15%)	1 (1.6%)	0.015	5 (14.3%)	0	0.054
Gastric cancer, *n* (%)	4 (4%)	4 (10%)	0	0.022	3 (8.6%)	0	0.239
Esophageal cancer, *n* (%)	4 (4%)	4 (10%)	0	0.022	4 (11.4%)	0	0.114
Urothelial cancer, *n* (%)	4 (4%)	4 (10%)	0	0.022	2 (5.7%)	0	0.493
Acute pancreatitis, *n* (%)	2 (2%)	2 (5%)	0	0.154	2 (5.7%)	0	0.493
Prostate cancer, *n* (%)	4 (4%)	3 (7.5%)	1 (1.6%)	0.298	3 (8.6%)	1 (2.9%)	0.614
Renal cell carcinoma, *n* (%)	2 (2%)	1 (2.5%)	1 (1.7%)	1.000	1 (2.9%)	1 (2.9%)	1.000
Others, *n* (%)	6 (5.9%)	6 (15%)	0	—	6 (17.1%)	0	—

Abbreviations: IQR, interquartile range; N.A., not applicable; PDAC, pancreatic ductal adenocarcinoma; SD, standard deviation; UICC 8th, Union for International Cancer Control TNM Classification, 8th Edition. † For the PDAC group, age at the last CT scan obtained before the diagnostic (PDAC-confirming) scan; for controls, age at the last available CT scan.

**Table 2:** Multivariable linear regression of the PV change coefficient (*n* = 101, R^2^ = 0.236).

Variable	β	SE	95% CI	*p* Value
Group (PDAC vs. control)	−0.129	0.054	−0.234 to −0.024	0.018
Age	−0.002	0.002	−0.006 to 0.002	0.282
Sex (male vs. female)	0.039	0.047	−0.053 to 0.132	0.408
Diabetes mellitus (yes vs. no)	−0.118	0.086	−0.286 to 0.050	0.170
Baseline PV	−0.0005	0.001	−0.003 to 0.002	0.727
Number of CT examinations	0.009	0.004	0.001 to 0.018	0.036

**Table 3:** Sensitivity analysis across pre-diagnostic truncation thresholds.

Truncation Threshold	Method	PDAC n	Estimate	*p* Value (vs. Control)
None (0 days)	Subject-specific slope	40	mean = +0.051	0.887
None (0 days)	Linear mixed-effects model	40	β (time × group) = +0.019	0.553
6 months	Subject-specific slope	40	mean = −0.205	<0.0001
6 months	Linear mixed-effects model	40	β (time × group) = −0.093	0.00046
1 year (primary analysis)	Subject-specific slope	40	mean = −0.212	<0.0001
1 year (primary analysis)	Linear mixed-effects model	40	β (time × group) = −0.126	0.00009
2 years	Subject-specific slope	37	mean = −0.186	0.0002
2 years	Linear mixed-effects model	37	β (time × group) = −0.100	0.00043
3 years	Subject-specific slope	34	mean = −0.268	<0.0001
3 years	Linear mixed-effects model	34	β (time × group) = −0.120	0.00013

## Data Availability

The data supporting the findings of this study are available from the corresponding author upon reasonable request.

## Supplementary Materials

The following supporting information can be downloaded at: https://www.mdpi.com/article/10.3390/cancers18183062/s1, Figure S1: automated pancreas segmentation; Figure S2: representative longitudinal volume trajectory; Figure S3: agreement between unenhanced and contrast-enhanced CT; Figure S4: PV change coefficient by tumor location; Figure S5: pancreatic volume by age and sex; Figure S6: PV change coefficient versus age with exploratory threshold; Figure S7: volcano plots for the two lesion-versus-normal comparisons (FPPA-high, FPPA-invasive); Table S1: clinicopathological features of nine resected cases; Table S2: 20 most robustly differentially expressed transcripts significant in both the FPPA-high and FPPA-invasive comparisons; Table S3: full list of 131 transcripts significant in both the FPPA-high and FPPA-invasive comparisons; Table S4: Fisher’s exact enrichment of nCounter Tumor Signaling 360 panel gene-set categories among the FPPA-high, FPPA-invasive, and 131-gene concordant transcript sets; Table S5: Fisher’s exact enrichment of nCounter Tumor Signaling 360 panel gene-set categories among transcripts significantly altered between pooled PanIN epithelium and invasive epithelium (Section 3.14); Table S6: candidate reference (housekeeping) genes and their geNorm-derived expression-stability ranking used for nCounter normalization; Table S7: covariate balance (standardized mean differences) before and after propensity-score matching.
