# Supplementary material for: Accelerated Pancreatic Volume Loss as a Potential Pre-Diagnostic Imaging Biomarker for Pancreatic Cancer Risk Enrichment: An AI-Assisted Volumetric Study
Source: Cancers (Basel). 2026 Sep 21;18(18):3062. doi: 10.3390/cancers18183062 (PMC13605397; doi:10.3390/cancers18183062)
Supplement: Supplementary file 1 [file cancers-18-03062-s001.zip › cancers-4537792-supplementary.pdf]

Supplementary Materials

Accelerated pancreatic volume loss as a potential pre-diagnostic imaging biomarker for pancreatic cancer risk enrichment: an AI-assisted volumetric study

Jun Nakahodo et al.

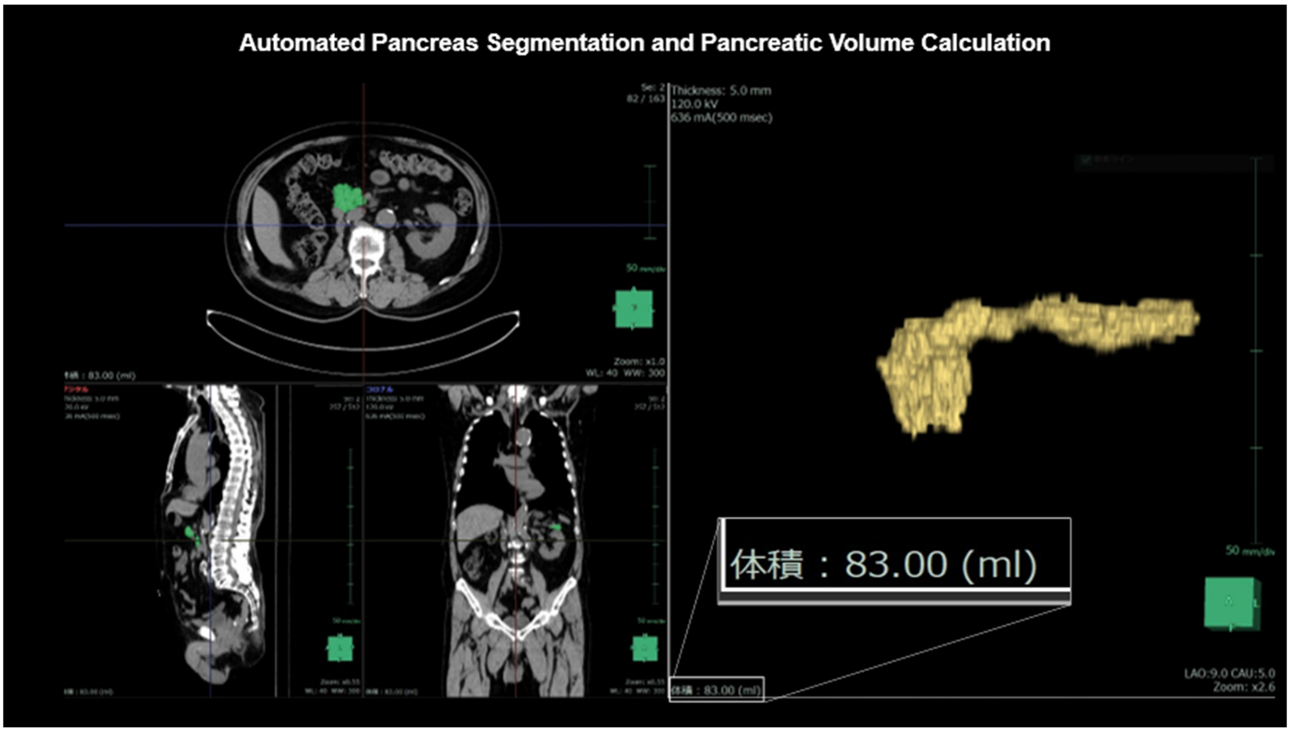

Supplementary Figure S1. Automated pancreas segmentation and pancreatic volume calculation using AI-assisted 3D analysis software.

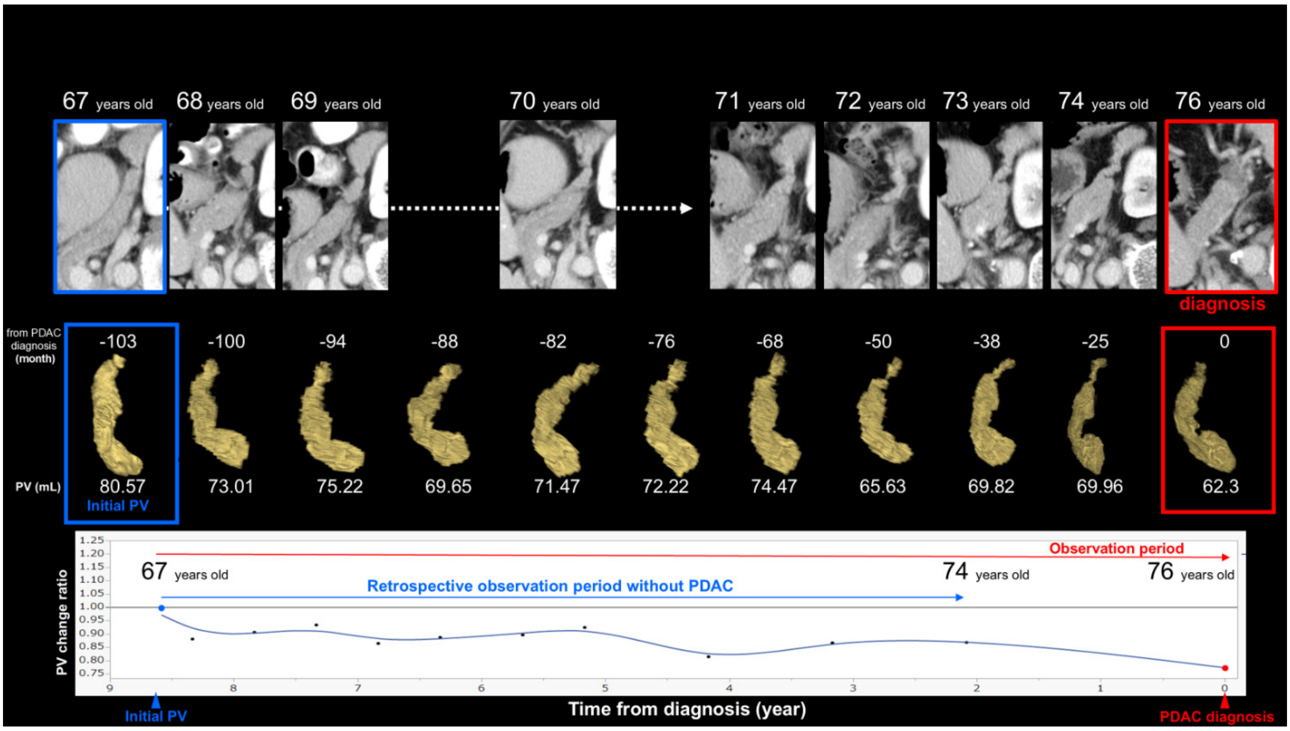

Supplementary Figure S2. Representative longitudinal pancreatic volume trajectory in a single patient over the 9 years preceding PDAC diagnosis, illustrating the definition of the initial PV and the observation window.

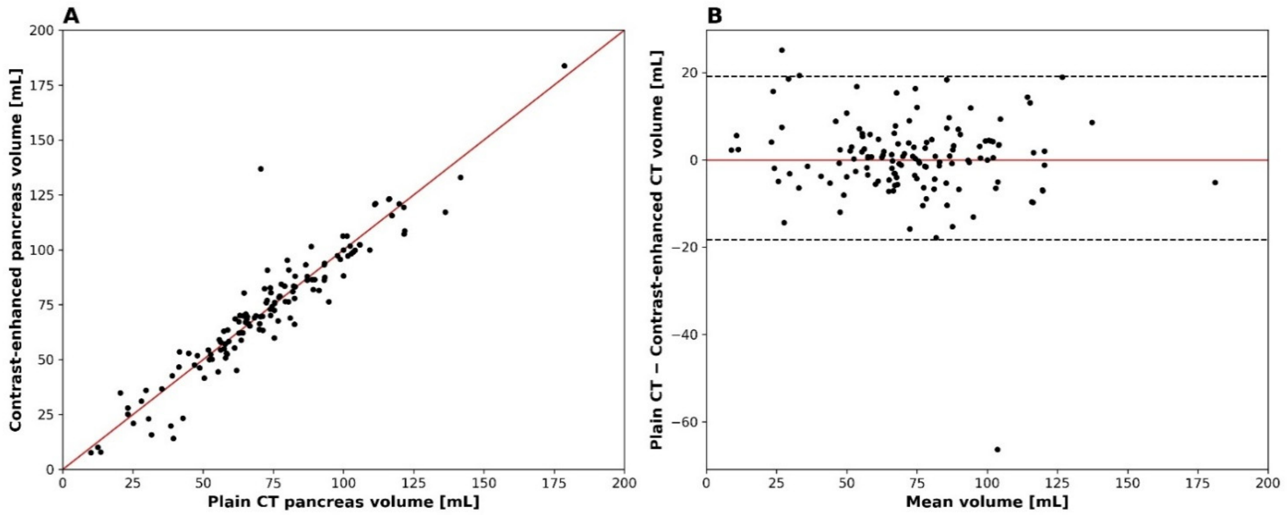

Supplementary Figure S3. Agreement of pancreatic volume measurements between unenhanced and contrast-enhanced CT (134 paired examinations). **(A)** Correlation plot. **(B)** Bland–Altman plot.

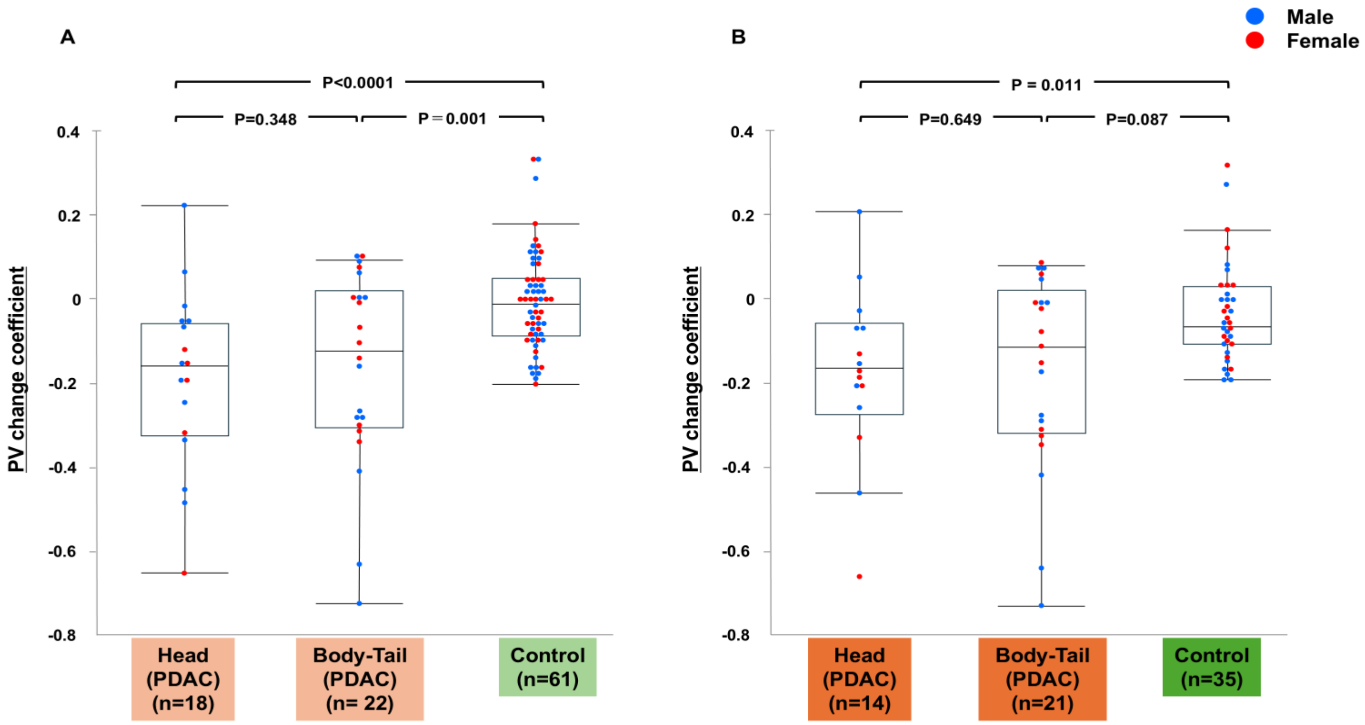

Supplementary Figure S4. PV change coefficient according to primary tumor location (head versus body-tail). (A) Full cohort. (B) Propensity-matched cohort.

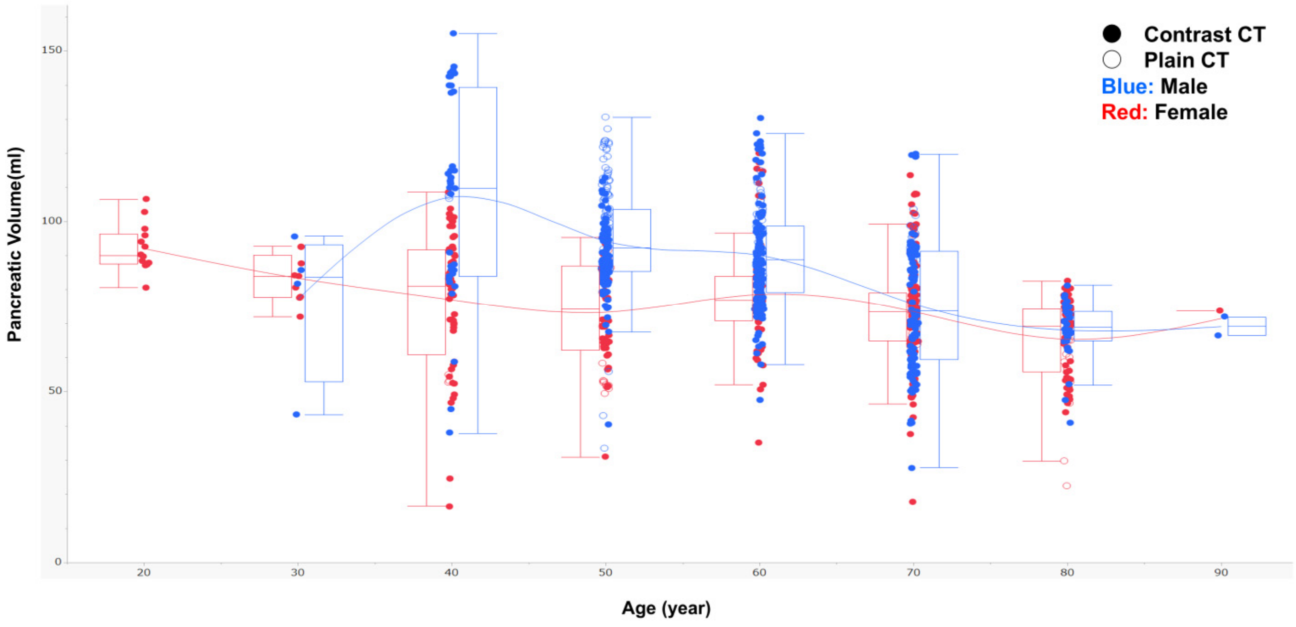

Supplementary Figure S5. Distribution of pancreatic volume according to age and sex among control patients.

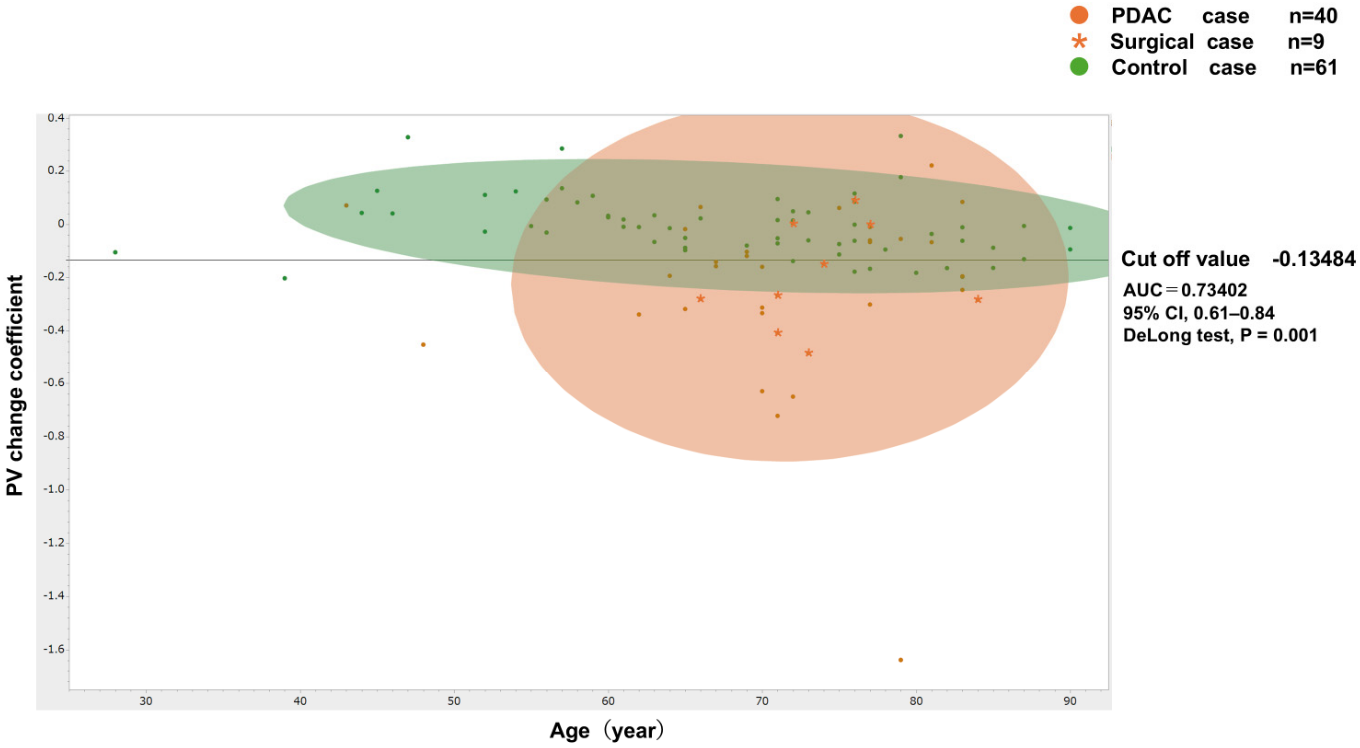

Supplementary Figure S6. Distribution of the PV change coefficient against age in patients with PDAC and controls, with the exploratory discriminative threshold indicated. Asterisks denote the nine surgically resected cases detailed in Supplementary Table S1.

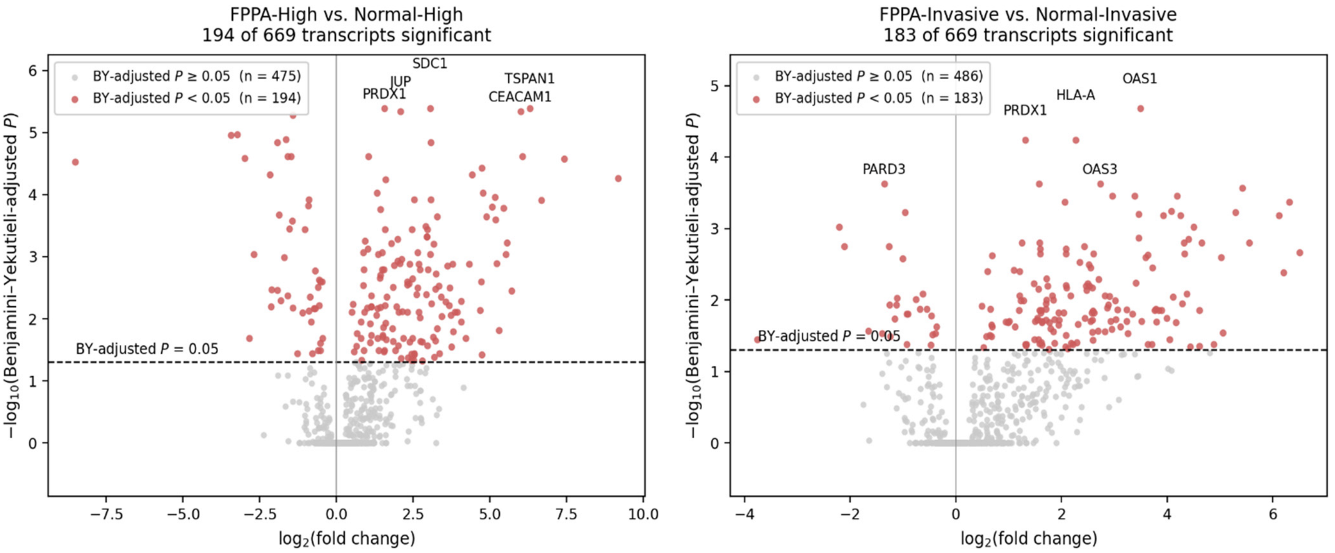

Supplementary Figure S7. Enlarged view of the volcano plots shown in Figure 7D, showing differential gene expression for the two lesion-versus-normal comparisons (FPPA-high vs. normal-high; FPPA-invasive vs. normal-invasive). The horizontal dashed line indicates the Benjamini–Yekutieli-adjusted significance threshold (adjusted P = 0.05). The five most significant transcripts in each comparison are labeled here, in addition to those labeled in Figure 7D.

Supplementary Tables

Supplementary Table S1. Clinicopathological features of the nine surgically resected PDAC cases

Variable	Case 1	Case 2	Case 3	Case 4	Case 5	Case 6	Case 7	Case 8	Case 9
Patient characteristics	Non-rapid volume loss	Non-rapid volume loss	Non-rapid volume loss	Rapid volume loss	Rapid volume loss	Rapid volume loss	Rapid volume loss	Rapid volume loss	Rapid volume loss
Age (years)	78	79	73	78	72	75	74	68	86
Sex	M	M	M	M	M	M	M	M	M
Pancreatic volumetry									
PV change coefficient (mL/month)	+0.091	−0.000	+0.004	−0.267	−0.408	−0.149	−0.484	−0.280	−0.283
Number of CT scans	6	8	26	7	7	14	16	12	3
Observation period (years)	5.8	6.5	11.4	6.0	5.2	6.5	6.6	6.4	5.1
Initial pancreatic volume (mL)	74.3	83.2	49.9	82.3	66.8	101.6	39.9	65.8	73.8
Tumor characteristics									
Tumor location	Body-tail	Body-tail	Body-tail	Body-tail	Body-tail	Head	Head	Body-tail	Body-tail
Tumor size (mm)	37	30	31	28	23	34	5	15	12
Pathological stage (UICC 8th)	IB	IB	IIB	IB	IIB	IB	IA	IA	IA
pT	2	2	2	2	2	2	1a	1c	1c
pN	0	0	1	0	1	0	0	0	0
Local invasion									
Lymphatic invasion (ly)	0	0	1	0	1	0	0	0	0
Venous invasion (v)	2	1	2	3	1	1	0	1	1
Neural invasion (ne)	2	1	1	0	1	1	0	3	2
Intraductal spread (mpd)	0	0	0	0	0	0	1	1	0
Histology									
Differentiation	tub2>por	wel>mod	mod>wel>por	wel>mod>por	por>mod	mod>wel>por	wel	wel>mod	wel>mod

PanIN assessment									
PanIN-positive slides (n)	0	9	6	8	13	3	8	9	4
Parenchymal slides (n)	5	19	10	11	15	3	8	12	4
PanIN prevalence (%)	0	47	60	73	87	100	100	75	100

Cases are ordered by rate of pancreatic volume change. Cases 1–3 showed non-rapid change and Cases 4–9 showed rapid volume loss, defined as a PV change coefficient below the exploratory threshold of −0.135 mL/month. PanIN prevalence was calculated as the proportion of PanIN-positive slides among all evaluable parenchymal slides, excluding areas of invasive carcinoma. Mean PanIN prevalence was 89.1% in the rapid volume loss group versus 35.8% in the non-rapid group ($P = 0.0181$). Cases 7–9 additionally underwent the exploratory molecular analysis described in Section 2.4 and Section 3.11, contributing the FPPA-invasive and normal-invasive regions (Figure 7; Supplementary Tables S2 and S3); the FPPA-high and normal-high regions used in the same analysis were obtained from three further patients with high-grade PanIN who are not included in this table.

Abbreviations: ly, lymphatic invasion; mod, moderately differentiated tubular adenocarcinoma; mpd, intraductal spread within the main pancreatic duct; ne, neural invasion; PanIN, pancreatic intraepithelial neoplasia; por, poorly differentiated adenocarcinoma; PV, pancreatic volume; tub2, moderately differentiated tubular adenocarcinoma; v, venous invasion; wel, well-differentiated tubular adenocarcinoma.

Supplementary Table S2. The 20 most robustly differentially expressed transcripts among those significant in both the FPPA-high-versus-normal-high and FPPA-invasive-versus-normal-invasive comparisons, ranked by the larger (less significant) of the two Benjamini–Yekutieli-adjusted P values

Gene	FPPA-High FC	FPPA-High 95% CI	FPPA-High BY-P	FPPA-Invasive FC	FPPA-Invasive 95% CI	FPPA-Invasive BY-P
OAS1	8.43	5.27–13.50	<0.0001	11.30	7.04–18.00	<0.0001
PRDX1	2.96	2.42–3.63	<0.0001	2.50	2.04–3.06	0.0001
PARD3	0.36	0.28–0.46	<0.0001	0.39	0.31–0.50	0.0002
PKM	3.01	2.28–3.98	0.0001	2.99	2.26–3.95	0.0002
OAS3	5.76	3.58–9.25	0.0001	6.65	4.14–10.70	0.0002
UBE2C	66.60	25.10–177.00	<0.0001	42.90	16.10–114.00	0.0003
TSPAN1	78.80	36.30–171.00	<0.0001	18.20	8.38–39.50	0.0003
SFN	172.00	51.00–578.00	<0.0001	79.50	23.60–268.00	0.0004
SDC1	8.35	5.61–12.40	<0.0001	4.18	2.81–6.23	0.0004
OAS2	7.69	4.08–14.50	0.0005	10.50	5.57–19.80	0.0003
CEACAM1	64.00	28.50–144.00	<0.0001	16.90	7.53–38.00	0.0006
ATG2B	0.37	0.31–0.46	<0.0001	0.51	0.42–0.62	0.0006
FOXM1	43.90	15.20–126.00	0.0002	39.10	13.60–113.00	0.0006
IL2RG	8.99	4.46–18.10	0.0006	11.00	5.44–22.10	0.0006
CARD11	27.30	11.40–65.10	0.0001	19.00	7.96–45.50	0.0007
TPX2	102.00	29.00–360.00	0.0001	69.40	19.70–245.00	0.0007
HLA-A	2.82	2.00–3.96	0.0009	4.81	3.42–6.76	0.0001
TOP2A	35.90	13.80–93.50	0.0001	22.60	8.68–59.00	0.0010
P4HB	0.11	0.07–0.17	<0.0001	0.22	0.14–0.35	0.0010
LAMB3	21.20	9.92–45.40	<0.0001	11.00	5.13–23.50	0.0013

Estimates derive from the differential expression output of the nCounter Tumor Signaling 360 panel analysis (three FPPA-high and three normal-high regions for the FPPA-high comparison; three FPPA-invasive and three normal-invasive regions for the FPPA-invasive comparison). All 20 transcripts shown were independently significant (Benjamini–Yekutieli-adjusted $P < 0.05$) in both comparisons; transcripts are ranked by the larger (i.e., less significant) of the two adjusted P values. FC, fold change; CI, confidence interval. Confidence intervals are wide for transcripts with large fold changes, reflecting the limited precision obtainable from three samples per group.

Supplementary Table S3. The full set of 131 transcripts significantly differentially expressed (Benjamini–Yekutieli-adjusted P < 0.05) in both the FPPA-high-versus-normal-high and FPPA-invasive-versus-normal-invasive comparisons (Section 2.4), ranked by the larger (less significant) of the two adjusted P values. All 131 transcripts were concordant in direction of fold change between the two comparisons.

Gene	FPPA-High FC	FPPA-High BY-P	FPPA-Invasive FC	FPPA-Invasive BY-P
OAS1	8.43	<0.0001	11.30	<0.0001
PRDX1	2.96	<0.0001	2.50	0.0001
PARD3	0.36	<0.0001	0.39	0.0002
PKM	3.01	0.0001	2.99	0.0002
OAS3	5.76	0.0001	6.65	0.0002
UBE2C	66.60	<0.0001	42.90	0.0003
TSPAN1	78.80	<0.0001	18.20	0.0003
SFN	172.00	<0.0001	79.50	0.0004
SDC1	8.35	<0.0001	4.18	0.0004
OAS2	7.69	0.0005	10.50	0.0003
CEACAM1	64.00	<0.0001	16.90	0.0006
ATG2B	0.37	<0.0001	0.51	0.0006
FOXM1	43.90	0.0002	39.10	0.0006
IL2RG	8.99	0.0006	11.00	0.0006
CARD11	27.30	0.0001	19.00	0.0007
TPX2	102.00	0.0001	69.40	0.0007
HLA-A	2.82	0.0009	4.81	0.0001
TOP2A	35.90	0.0001	22.60	0.0010
P4HB	0.11	<0.0001	0.22	0.0010
LAMB3	21.20	<0.0001	11.00	0.0013
BUB1	29.40	0.0002	21.20	0.0014
JUP	4.27	<0.0001	2.38	0.0016
CDK1	33.70	0.0002	20.00	0.0016
MKI67	36.60	0.0003	25.00	0.0016

Gene	FPPA-High FC	FPPA-High BY-P	FPPA-Invasive FC	FPPA-Invasive BY-P
ICOS	37.40	0.0013	46.80	0.0016
SAV1	0.37	0.0003	0.42	0.0018
EIF4EBP1	0.12	<0.0001	0.23	0.0018
TNFSF13B	2.66	0.0022	3.01	0.0016
STING1	2.92	0.0016	3.04	0.0023
CD4	4.35	0.0011	4.23	0.0023
BLM	7.76	0.0004	6.10	0.0023
SOD1	2.05	<0.0001	1.61	0.0024
LAMC2	12.20	0.0014	12.10	0.0025
IDO1	26.40	0.0025	32.30	0.0025
IGF1R	0.32	<0.0001	0.50	0.0026
IL7	5.56	0.0022	5.74	0.0032
KRT17	52.30	0.0035	91.00	0.0022
CD3E	6.44	0.0013	5.87	0.0036
PLCB3	2.71	0.0002	2.15	0.0038
SERPINB5	577.00	0.0001	73.70	0.0041
STK4	2.19	0.0042	2.32	0.0040
PDCD1LG2	10.60	0.0044	13.20	0.0036
LTBP1	3.66	0.0014	3.30	0.0050
TGM2	6.10	0.0041	6.52	0.0052
LAG3	8.52	0.0057	12.50	0.0023
LCK	13.80	0.0013	10.70	0.0058
NCK1	1.45	0.0058	1.52	0.0040
PTGER2	6.57	0.0016	5.65	0.0059
PLA2G4A	3.45	0.0006	2.78	0.0059
CGAS	3.98	0.0012	3.35	0.0063

Gene	FPPA-High FC	FPPA-High BY-P	FPPA-Invasive FC	FPPA-Invasive BY-P
MST1R	8.41	0.0001	4.30	0.0068
ITGA8	8.36	0.0009	6.00	0.0068
SLC2A1	4.96	0.0077	5.56	0.0074
CCNB2	47.10	0.0006	20.70	0.0082
MLST8	0.53	0.0001	0.65	0.0082
CCNF	5.12	0.0027	4.49	0.0087
CCNB1	5.50	0.0004	3.54	0.0087
ITGB6	26.80	<0.0001	7.07	0.0087
CXCL10	15.20	0.0078	17.20	0.0090
TNFSF11	10.70	0.0091	20.10	0.0023
ATXN7	0.35	0.0004	0.46	0.0095
PRKCB	6.57	0.0095	7.52	0.0089
ATF4	0.49	0.0004	0.59	0.0097
ITK	10.00	0.0030	8.11	0.0102
CCR4	16.70	0.0111	19.50	0.0112
EIF2AK3	0.27	0.0002	0.42	0.0117
HLA-E	2.13	0.0121	2.31	0.0087
CDK12	1.41	0.0078	1.41	0.0122
CD3D	9.32	0.0033	7.31	0.0128
BAX	1.83	0.0009	1.57	0.0134
RB1CC1	0.61	0.0069	0.62	0.0134
FASLG	14.50	0.0077	13.80	0.0134
MAP2K2	0.54	0.0001	0.68	0.0134
CD3G	13.00	0.0032	9.41	0.0137
ITGA1	2.88	0.0138	3.02	0.0138
ITGB5	2.81	0.0016	2.29	0.0139

Gene	FPPA-High FC	FPPA-High BY-P	FPPA-Invasive FC	FPPA-Invasive BY-P
PSMB10	3.00	0.0117	3.07	0.0139
IL7R	6.41	0.0077	6.01	0.0141
ENTPD1	3.53	0.0143	5.08	0.0030
PARP3	7.45	0.0003	3.83	0.0151
SLPI	7.61	0.0041	5.94	0.0151
CHEK1	7.71	0.0005	4.13	0.0151
MS4A2	20.10	0.0013	10.10	0.0157
SRM	0.27	<0.0001	0.53	0.0158
BRAF	0.67	0.0024	0.73	0.0165
SHH	45.80	0.0009	15.90	0.0180
FGFR1	0.22	<0.0001	0.45	0.0184
B2M	2.19	0.0185	3.04	0.0019
ITPR3	4.05	0.0078	3.66	0.0186
FANCI	4.18	0.0013	2.90	0.0189
ZEB1	2.83	0.0195	3.30	0.0100
GPX1	2.19	0.0064	2.01	0.0197
CD40LG	12.70	0.0060	9.41	0.0197
BIRC5	16.60	0.0052	11.50	0.0197
HMGA1	3.58	0.0031	2.82	0.0197
MYD88	2.18	0.0063	1.98	0.0206
ITGAL	5.59	0.0206	5.98	0.0189
CCR7	18.50	0.0206	24.40	0.0138
CXCL9	25.40	0.0073	18.00	0.0207
NBL1	3.59	0.0232	3.86	0.0189
HSD11B1	6.90	0.0238	7.60	0.0197
CCR5	5.21	0.0238	6.15	0.0151

Gene	FPPA-High FC	FPPA-High BY-P	FPPA-Invasive FC	FPPA-Invasive BY-P
CD28	8.84	0.0195	8.62	0.0238
GMPS	0.72	0.0025	0.78	0.0238
PRKCA	1.83	0.0029	1.60	0.0238
HLA-C	2.12	0.0243	2.44	0.0102
PCLAF	6.25	0.0009	3.46	0.0250
GZMK	11.70	0.0053	7.72	0.0257
MYC	0.23	0.0034	0.32	0.0271
CCL5	4.41	0.0077	3.60	0.0279
SMC3	0.69	0.0025	0.76	0.0295
RAD51AP1	13.20	0.0088	9.56	0.0295
PDPK1	0.73	0.0206	0.74	0.0303
BCL2L1	1.89	0.0006	1.49	0.0323
ERN1	0.33	0.0042	0.42	0.0324
TAP1	2.97	0.0325	4.22	0.0062
HGF	2.59	0.0326	2.90	0.0180
PSMB9	3.79	0.0327	5.79	0.0063
LMNB1	2.92	0.0342	2.95	0.0356
EGF	0.00	<0.0001	0.07	0.0357
PRDX2	0.58	0.0361	0.53	0.0158
SELL	5.78	0.0366	6.47	0.0271
BRCA2	6.65	0.0228	6.03	0.0367
FAS	5.40	0.0370	5.97	0.0280
IL2RB	5.83	0.0380	8.33	0.0137
TIGIT	9.12	0.0414	14.30	0.0140
KLRB1	9.53	0.0019	4.66	0.0420
BCAP31	0.61	0.0017	0.72	0.0425

Gene	FPPA-High FC	FPPA-High BY-P	FPPA-Invasive FC	FPPA-Invasive BY-P
TNFRSF4	5.00	0.0428	7.43	0.0117
CCR2	4.28	0.0429	5.44	0.0180
MSLN	39.50	0.0155	24.50	0.0444

FC, linear fold change relative to normal parenchyma; BY-P, Benjamini–Yekutieli-adjusted P value. All values derive from the differential expression output of the nCounter Tumor Signaling 360 panel analysis for the FPPA-high-versus-normal-high and FPPA-invasive-versus-normal-invasive comparisons. All 131 transcripts were concordant in direction of fold change between the two comparisons.

Supplementary Table S4. Fisher's exact enrichment of nCounter Tumor Signaling 360 panel gene-set categories among differentially expressed transcripts in the FPPA-high-versus-normal-high and FPPA-invasive-versus-normal-invasive comparisons (Section 2.4), and among the 131 transcripts concordant across both comparisons (Section 3.11). Categories with a raw (unadjusted) Fisher's exact P < 0.05 in at least one comparison are shown, ranked by comparison and then by P value.

Comparison	Annotation Type	Category	N in category (of 669)	N significant	% significant	Odds ratio	Fisher exact P	BH-adjusted P
FPPA-high vs. normal-high	Functional category	Antigen Presentation	15	9	60.0	3.80	0.0112	0.2886
FPPA-high vs. normal-high	Functional category	DNA Damage Repair	33	16	48.5	2.42	0.0120	0.2886
FPPA-high vs. normal-high	Functional category	Apoptosis	12	7	58.3	3.52	0.0310	0.3530
FPPA-high vs. normal-high	Functional category	Chemokine Signaling	20	10	50.0	2.53	0.0362	0.3530
FPPA-high vs. normal-high	Functional category	Cell Cycle	57	23	40.4	1.74	0.0368	0.3530
FPPA-high vs. normal-high	Hallmark theme	Evading Growth Suppressors	57	23	40.4	1.74	0.0368	0.2110
FPPA-invasive vs. normal-invasive	Hallmark theme	Avoiding Immune Destruction	97	49	50.5	3.34	1.16e-07	1.16e-06
FPPA-invasive vs. normal-invasive	Functional category	Antigen Presentation	15	12	80.0	11.30	2.68e-05	0.0013
FPPA-invasive vs. normal-invasive	Functional category	T-cell Costimulation	18	12	66.7	5.61	4.80e-04	0.0115
FPPA-invasive vs. normal-invasive	Functional category	TCR Signaling	30	16	53.3	3.23	0.0018	0.0261
FPPA-invasive vs. normal-invasive	Functional category	Interferon Response	33	17	51.5	3.01	0.0022	0.0261
FPPA-invasive vs. normal-invasive	Functional category	TNF Superfamily	11	7	63.6	4.79	0.0121	0.1165
FPPA-invasive vs. normal-invasive	Functional category	B-cell Function	5	4	80.0	10.84	0.0214	0.1716
FPPA-invasive vs. normal-invasive	Hallmark theme	Resisting Cell Death	21	10	47.6	2.50	0.0354	0.1237
FPPA-invasive vs. normal-invasive	Hallmark theme	Tumor-promoting Inflammation	163	54	33.1	1.45	0.0371	0.1237
Concordant (131 genes)	Hallmark theme	Avoiding Immune Destruction	97	32	33.0	2.35	4.88e-04	0.0049
Concordant (131 genes)	Functional category	Antigen Presentation	15	9	60.0	6.54	5.68e-04	0.0273
Concordant (131 genes)	Functional category	TCR Signaling	30	11	36.7	2.50	0.0196	0.4325
Concordant (131 genes)	Hallmark theme	Resisting Cell Death	21	8	38.1	2.63	0.0360	0.1800

Comparison	Annotation Type	Category	N in category (of 669)	N significant	% significant	Odds ratio	Fisher exact P	BH-adjusted P
Concordant (131 genes)	Functional category	Interferon Response	33	11	33.3	2.15	0.0403	0.4325
Concordant (131 genes)	Functional category	T-cell Costimulation	18	7	38.9	2.70	0.0440	0.4325
Concordant (131 genes)	Functional category	TNF Superfamily	11	5	45.5	3.52	0.0451	0.4325

N in category, number of the 669 panel genes tested for differential expression that belong to the indicated functional category or Hallmark-of-Cancer theme, per the panel's gene-set annotation reference file (LBL-10779-01); N significant, number of those category genes with Benjamini–Yekutieli-adjusted $P < 0.05$ in the indicated comparison; Odds ratio and Fisher exact P, from a one-sided Fisher's exact test (category membership vs. differential-expression significance, using all 669 panel genes as the background); BH-adjusted P, Benjamini–Hochberg-adjusted P value across all 48 functional categories and 10 Hallmark themes tested within each comparison. No category reached BH-adjusted $P < 0.05$ in the FPPA-high-versus-normal-high comparison.

Supplementary Table S5. Fisher's exact enrichment of nCounter Tumor Signaling 360 panel gene-set categories among the five transcripts significantly altered (Benjamini–Yekutieli-adjusted P < 0.05) between the pooled PanIN-epithelium group (low-grade and high-grade PanIN, nine regions from six patients) and the invasive-epithelium group (three regions, Section 3.14). Categories with a raw (unadjusted) Fisher's exact P < 0.05 are shown.

Comparison	Annotation Type	Category	N in category (of 669)	N significant	Odds ratio	Fisher exact P	BH-adjusted P
PanIN vs. Invasion (epithelium)	Functional category	B-cell Function	5	3	496.50	2.00e-06	9.60e-05
PanIN vs. Invasion (epithelium)	Hallmark theme	Avoiding Immune Destruction	97	3	9.10	0.0237	0.2375

N in category, number of the 669 panel genes tested for differential expression that belong to the indicated functional category or Hallmark-of-Cancer theme, per the panel's gene-set annotation reference file (LBL-10779-01); N significant, number of those category genes among the five transcripts significant in the PanIN-versus-invasion epithelial comparison (Section 3.14); Odds ratio and Fisher exact P, from a one-sided Fisher's exact test (category membership vs. differential-expression significance, using all 669 panel genes as the background); BH-adjusted P, Benjamini–Hochberg-adjusted P value across all 48 functional categories and 10 Hallmark themes tested. Only B-cell Function remained significant after this correction.

Supplementary Table S6. Candidate reference (housekeeping) genes ranked by expression stability (geNorm) and used for nCounter normalization

Gene	geNorm stability rank	SD after normalization	Status
TMUB2	1	0.167	Selected
ERCC3	2	0.145	Selected
NRDE2	3	0.181	Selected
PUM1	4	0.111	Selected
SF3A1	5	0.123	Selected
TLK2	6	0.196	Selected
ABCF1	7	0.163	Selected
SDHA	8	0.189	Selected
TBP	9	0.201	Selected
MRPL19	10	0.253	Selected
GUSB	11	0.279	Selected
TBC1D10B	12	0.261	Selected
STK11IP	13	0.277	Selected
POLR2A	14	0.280	Selected
G6PD	15	0.314	Selected
PSMC4	16	0.336	Selected
DNAJC14	17	0.340	Selected
UBB	—	0.624	Discarded
TFRC	—	0.400	Discarded
OAZ1	—	0.479	Discarded

Candidate reference genes provided with the nCounter Tumor Signaling 360 panel were ranked for expression stability across the profiled regions using the geNorm algorithm. The 17 most stable candidates (ranks 1–17) were retained and their geometric mean used for reference-gene normalization; three candidates with high residual variability (SD after normalization ≥ 0.4) were discarded. SD, standard deviation of normalized expression across samples.

Supplementary Table S7. Covariate balance before and after propensity-score matching, expressed as standardized mean differences (SMDs).

**Covariate**	**SMD, full cohort (before matching)**	**SMD, matched cohort (after matching)**
Age at last CT scan	+0.59	−0.05
Sex, male	+0.12	+0.00
Number of CT scans	-1.10	-0.99
Observation period (months)	-0.22	-0.19
Initial pancreatic volume (mL)	-0.40	-0.16
Diabetes mellitus	+0.41	+0.33
Colon cancer	+0.62	+0.49
Breast cancer	+0.27	-0.25
Lung cancer	+0.50	+0.58
Gastric cancer	+0.47	+0.43
Esophageal cancer	+0.47	+0.51
Urothelial cancer	+0.47	+0.35
Acute pancreatitis	+0.32	+0.35
Prostate cancer	+0.29	+0.25
Renal cell carcinoma	+0.06	+0.00
Others	+0.59	+0.64

SMD, standardized mean difference (PDAC minus control), computed from the descriptive statistics in Table 1 (means ± standard deviation for continuous variables; proportions for categorical variables), except that the age standardized mean differences (before and after matching) were computed from individual-level patient data, because age is summarized as median (IQR) in Table 1; |SMD| < 0.10 indicates adequate balance. Age at last CT scan and sex were the propensity-score matching variables. Dermatologic malignancy—the control-group selection criterion (0% in the PDAC group vs. 100% in controls)—is not expressed as a standardized mean difference. After matching, age and sex were balanced, whereas the number of CT scans and several comorbidities retained |SMD| > 0.10; this residual imbalance—most notably in the number of CT scans—was addressed analytically by multivariable adjustment and by the linear mixed-effects model, which uses each patient's own longitudinal trajectory.
